# Supplementary material for: Dynalign II: common secondary structure prediction for RNA homologs with domain insertions
Source: Nucleic Acids Res. 2014 Nov 21;42(22):13939–48. doi: 10.1093/nar/gku1172 (PMC4267632; doi:10.1093/nar/gku1172)
Supplement: SUPPLEMENTARY DATA [file supp_gku1172_nar-02021-z-2014-File012.zip › manual/javadoc/index-all.html]

Index


JavaScript is disabled on your browser.


- Overview
- Package
- Class
- Tree
- Index
- Help

- Prev
- Next

- Frames
- No Frames

- All Classes

A B C D E F G H I J L M N O P R S T V W Z 


## A

actionPerformed(ActionEvent) - Method in class RNAstructure\_java\_interface.source.windows.InternalWindow


actionPerformed(ActionEvent) - Method in class RNAstructure\_java\_utilities.RNAstructureMenu


activate() - Method in class RNAstructure\_java\_interface.source.windows.MultilignWindow


activate() - Method in class RNAstructure\_java\_interface.source.windows.MultiWindow
:   Activate data input for this window.

activate() - Method in class RNAstructure\_java\_interface.source.windows.TurboFoldWindow


activateMultilign() - Method in class RNAstructure\_java\_interface.source.startup.RNAstructureBackendCalculator


activateTurboFold() - Method in class RNAstructure\_java\_interface.source.startup.RNAstructureBackendCalculator


addAnnotationProbability(String) - Method in class RNAstructure\_java\_drawing.source.proxy.StructureBackend


addAnnotationSHAPE(String) - Method in class RNAstructure\_java\_drawing.source.proxy.StructureBackend


addBackendTuple(String, String) - Method in class RNAstructure\_java\_interface.source.windows.MultilignWindow


addBackendTuple(String, String) - Method in class RNAstructure\_java\_interface.source.windows.MultiWindow
:   Add a tuple to the calculation back end.

addBackendTuple(String, String) - Method in class RNAstructure\_java\_interface.source.windows.TurboFoldWindow


addCheckBoxMenuItem(String, String) - Method in class RNAstructure\_java\_utilities.RNAstructureMenu
:   Add a check box menu item with rollover text.

addClosingListener(JDialog) - Method in class RNAstructure\_java\_drawing.source.runners.RunnerTemplate
:   Add a listener to the dialog spawned by this template that exits the
    application when the dialog is closed.

addGeneralSection() - Method in class RNAstructure\_java\_interface.source.menus.ConstraintsMenu
:   Add a menu section of general constraints commands.

addMaxPairingDistanceSection() - Method in class RNAstructure\_java\_interface.source.menus.ConstraintsMenu
:   Add a menu section that handles maximum pairing distance.

addMenuItem(String, String) - Method in class RNAstructure\_java\_utilities.RNAstructureMenu
:   Add a plain text menu item with rollover text.

addMenuItem(String, String, char) - Method in class RNAstructure\_java\_utilities.RNAstructureMenu
:   Add a plain text menu item with rollover text and a key stroke which
    can activate it.

addMenuItem(String, String, String) - Method in class RNAstructure\_java\_utilities.RNAstructureMenu
:   Add a plain text menu item with rollover text and a key stroke which
    can activate it.

addMultilignTuple(String, String) - Method in class RNAstructure\_java\_interface.source.startup.RNAstructureBackendCalculator


addSaveRestoreSection() - Method in class RNAstructure\_java\_interface.source.menus.ConstraintsMenu
:   Add a menu section that handles saving and restoring constraints.

addSHAPESection() - Method in class RNAstructure\_java\_interface.source.menus.ConstraintsMenu
:   Add a menu section that handles SHAPE constraints.

addShowResetSection() - Method in class RNAstructure\_java\_interface.source.menus.ConstraintsMenu
:   Add a menu section that handles showing and resetting constraints.

addTurboFoldTuple(String, String) - Method in class RNAstructure\_java\_interface.source.startup.RNAstructureBackendCalculator


ALWAYS - Static variable in class RNAstructure\_java\_utilities.ScrollerPane
:   A generic constant symbolizing that a scroll bar must always be used.

AnnotationMenu - Class in RNAstructure\_java\_drawing.source.menus
:   A class that handles a menu which can annotate a drawn structure.

AnnotationMenu(StructureDialog) - Constructor for class RNAstructure\_java\_drawing.source.menus.AnnotationMenu
:   Constructor.

ApplicationRootFrame - Class in RNAstructure\_java\_interface.source.startup
:   A class that creates the application frame of the RNAstructure GUI.

ApplicationRootFrame() - Constructor for class RNAstructure\_java\_interface.source.startup.ApplicationRootFrame
:   Constructor.

areas - Variable in class RNAstructure\_java\_interface.source.windows.SequenceDisplayWindow
:   The array of text areas in this window.


## B

backend - Variable in class RNAstructure\_java\_drawing.source.dialogs.PlotDialog
:   The back end object that reads data for dot plots.

backend - Variable in class RNAstructure\_java\_drawing.source.dialogs.StructureDialog
:   The back end object that reads data for structures.

backend - Variable in class RNAstructure\_java\_interface.source.menus.ConstraintsMenu
:   The back end calculator that's linked to this menu.

backend - Variable in class RNAstructure\_java\_interface.source.windows.InternalWindow
:   The back end calculator that's linked to this window.

bar - Variable in class RNAstructure\_java\_interface.source.startup.ApplicationRootFrame
:   The main RNAstructure toolbar.

BorderBuilder - Class in RNAstructure\_java\_utilities
:   A class that places a border type on a specified component type.

BorderBuilder() - Constructor for class RNAstructure\_java\_utilities.BorderBuilder


BorderedButtonPanel - Class in RNAstructure\_java\_utilities
:   A class that produces a panel which contains a button with a border
    around it.

BorderedButtonPanel(ActionListener, String) - Constructor for class RNAstructure\_java\_utilities.BorderedButtonPanel
:   Text Constructor.

BorderedButtonPanel(ActionListener, String, int) - Constructor for class RNAstructure\_java\_utilities.BorderedButtonPanel
:   Border Constructor.

buildAllSubDataStructure(String, boolean) - Method in class RNAstructure\_java\_interface.source.startup.RNAstructureBackendCalculator


buildBackPanel() - Method in class RNAstructure\_java\_interface.source.startup.ApplicationRootFrame
:   Create the gray back panel that fills the main frame.

buildBifoldDataStructure(String, String, boolean) - Method in class RNAstructure\_java\_interface.source.startup.RNAstructureBackendCalculator


buildBipartitionDataStructure(String, String, boolean) - Method in class RNAstructure\_java\_interface.source.startup.RNAstructureBackendCalculator


buildButtonPanel() - Method in class RNAstructure\_java\_interface.source.windows.SequenceDisplayWindow
:   Make the button panel at the bottom of the window.

buildDialog(String, NumberField...) - Method in class RNAstructure\_java\_utilities.ValueSelectionDialog
:   Build the value selection dialog.

buildDynalignAlignmentMenu() - Method in class RNAstructure\_java\_interface.source.menus.ConstraintsMenu
:   Build a complete menu that handles Dynalign alignment constraints.

buildDynalignDataStructure(String, String, boolean) - Method in class RNAstructure\_java\_interface.source.startup.RNAstructureBackendCalculator


buildEfn2DataStructure(String, boolean) - Method in class RNAstructure\_java\_interface.source.startup.RNAstructureBackendCalculator


buildFoldDataStructure(String, boolean) - Method in class RNAstructure\_java\_interface.source.startup.RNAstructureBackendCalculator


buildInfoLabel() - Method in class RNAstructure\_java\_interface.source.startup.ApplicationRootFrame
:   Create the label at the bottom of the main frame.

buildInputRegion(int, String) - Method in class RNAstructure\_java\_interface.source.windows.SequenceDisplayWindow
:   Build a text input region for this window.

buildLegend(String...) - Method in class RNAstructure\_java\_drawing.source.dialogs.ImageDialog
:   Build the legend grid for this panel.

buildMaxExpectDataStructure(String) - Method in class RNAstructure\_java\_interface.source.startup.RNAstructureBackendCalculator


buildMaxLoopMenu() - Method in class RNAstructure\_java\_interface.source.menus.ConstraintsMenu
:   Build a complete menu that handles maximum loop size.

buildOligoScreenDataStructure() - Method in class RNAstructure\_java\_interface.source.startup.RNAstructureBackendCalculator


buildOligoWalkDataStructure(String, boolean) - Method in class RNAstructure\_java\_interface.source.startup.RNAstructureBackendCalculator


buildPartitionDataStructure(String, boolean) - Method in class RNAstructure\_java\_interface.source.startup.RNAstructureBackendCalculator


buildProbKnotDataStructure(String) - Method in class RNAstructure\_java\_interface.source.startup.RNAstructureBackendCalculator


buildRefoldDynalignDataStructure(String) - Method in class RNAstructure\_java\_interface.source.startup.RNAstructureBackendCalculator


buildRefoldSingleDataStructure(String) - Method in class RNAstructure\_java\_interface.source.startup.RNAstructureBackendCalculator


buildRemovePseudoknotsDataStructure(String, boolean) - Method in class RNAstructure\_java\_interface.source.startup.RNAstructureBackendCalculator


buildResultsWindow(String, int, int, int) - Method in class RNAstructure\_java\_interface.source.windows.OligoWalkWindow
:   Build the results window for this OligoWalk calculation.

buildSpecificOptionsPanel() - Method in class RNAstructure\_java\_interface.source.windows.MultilignWindow


buildSpecificOptionsPanel() - Method in class RNAstructure\_java\_interface.source.windows.MultiWindow
:   Build an input options box specific to this window.

buildSpecificOptionsPanel() - Method in class RNAstructure\_java\_interface.source.windows.TurboFoldWindow


buildStochasticDataStructure(String) - Method in class RNAstructure\_java\_interface.source.startup.RNAstructureBackendCalculator


buildTemperatureMenu() - Method in class RNAstructure\_java\_interface.source.menus.ConstraintsMenu
:   Build a complete menu that handles temperature.

buildToolBar() - Method in class RNAstructure\_java\_interface.source.startup.ApplicationRootFrame
:   Build the main RNAstructure toolbar.

buildUnimolecularMenu() - Method in class RNAstructure\_java\_interface.source.menus.ConstraintsMenu
:   Build a complete menu that handles if unimolecular pairs are allowed.


## C

canFoldOligoOligo(int) - Method in class RNAstructure\_java\_interface.source.startup.RNAstructureBackendCalculator


canFoldOligoSelf(int) - Method in class RNAstructure\_java\_interface.source.startup.RNAstructureBackendCalculator


cards - Variable in class RNAstructure\_java\_interface.source.windows.TurboFoldWindow
:   The layout that holds the dynamic options applicable to a mode.

checkOS() - Static method in class RNAstructure\_java\_utilities.MacChecker
:   Check the OS to see if it's a Mac, and if it is, set a few system
    properties to make things more "Mac-like."

checkValue() - Method in class RNAstructure\_java\_utilities.NumberField
:   Check the value of a field to make sure it's valid.

checkValue() - Method in class RNAstructure\_java\_utilities.NumberField.DoubleField


checkValue() - Method in class RNAstructure\_java\_utilities.NumberField.FloatField


checkValue() - Method in class RNAstructure\_java\_utilities.NumberField.IntegerField


CIRCLED\_NUCS - Static variable in class RNAstructure\_java\_drawing.source.dialogs.StructureDialog
:   Boolean that tells whether a structure's nucleotides are circled.

clearAnnotation() - Method in class RNAstructure\_java\_drawing.source.dialogs.StructureDialog
:   Clear annotation from this dialog.

clearDynalignAlignmentConstraints() - Method in class RNAstructure\_java\_interface.source.startup.RNAstructureBackendCalculator


clearFoldingConstraints(int) - Method in class RNAstructure\_java\_interface.source.startup.RNAstructureBackendCalculator


CLOCKWISE\_STRUCTURE - Static variable in class RNAstructure\_java\_drawing.source.dialogs.StructureDialog
:   Boolean that tells whether a structure is rendered clockwise.

columnWidth - Variable in class RNAstructure\_java\_interface.source.windows.MultiWindow
:   The width of each column of the window (input controls in column 1,
    sequence set in column 2).

combineFileNames(String, String, String) - Method in class RNAstructure\_java\_interface.source.windows.ModuleWindow
:   Combine two file names into a new file name.

components - Variable in class RNAstructure\_java\_utilities.FieldPanel
:   The components on this panel (buttons or labels).

constraints - Variable in class RNAstructure\_java\_interface.source.windows.ModuleWindow
:   The GridBagConstraints for this window.

ConstraintsMenu - Class in RNAstructure\_java\_interface.source.menus
:   A class that creates a folding constraints menu.

ConstraintsMenu(RNAstructureBackendCalculator) - Constructor for class RNAstructure\_java\_interface.source.menus.ConstraintsMenu
:   General Constructor.

ConstraintsMenu.NucOrHelixDialog - Class in RNAstructure\_java\_interface.source.menus
:   An inner class which creates a dialog that sets constraints on single
    nucleotides or helices.

ConstraintsMenu.NucOrHelixDialog(String, String) - Constructor for class RNAstructure\_java\_interface.source.menus.ConstraintsMenu.NucOrHelixDialog
:   Constructor.

convertToolTips() - Method in class RNAstructure\_java\_interface.source.menus.RolloverMenu
:   Convert tooltips to rollover text.

createAdditionalControls() - Method in class RNAstructure\_java\_drawing.source.menus.DotsMenu.RangeDialog


createAdditionalControls() - Method in class RNAstructure\_java\_interface.source.windows.OligoWalkWindow.OligoSelectionDialog


createAdditionalControls() - Method in class RNAstructure\_java\_utilities.ValueSelectionDialog
:   Create any additional controls that need to show up on this dialog.

createDefaultMenuBar(RNAstructureMenu...) - Method in class RNAstructure\_java\_drawing.source.dialogs.ImageDialog
:   Create the image dialog's default menu bar.

createDisabled(String) - Static method in class RNAstructure\_java\_utilities.FileField
:   Create a disabled file input field.

createDNA() - Static method in class RNAstructure\_java\_interface.source.menus.NucleicAcidMenu
:   Create a DNA menu.

createDrawnImage(Graphics2D) - Method in class RNAstructure\_java\_drawing.source.dialogs.ImageDialog
:   Create the image drawn on this panel.

createDrawnImage(Graphics2D) - Method in class RNAstructure\_java\_drawing.source.dialogs.PlotDialog


createDrawnImage(Graphics2D) - Method in class RNAstructure\_java\_drawing.source.dialogs.StructureDialog


createEmptyBox(String) - Static method in class RNAstructure\_java\_utilities.HTMLCheckBox
:   Create an empty check box.

createEnabled(String) - Static method in class RNAstructure\_java\_utilities.FileField
:   Create an enabled file input field.

createInputPanel() - Method in class RNAstructure\_java\_interface.source.windows.OligoWalkWindow
:   Create the panel which holds input controls.

createResultsPanel() - Method in class RNAstructure\_java\_interface.source.windows.OligoWalkWindow
:   Create the panel which holds results data.

createRNA() - Static method in class RNAstructure\_java\_interface.source.menus.NucleicAcidMenu
:   Create a RNA menu.

createSelectedBox(String) - Static method in class RNAstructure\_java\_utilities.HTMLCheckBox
:   Create a selected check box.

createSelectionAction() - Method in class RNAstructure\_java\_drawing.source.dialogs.ImageDialog.ZoomDialog


createSelectionAction() - Method in class RNAstructure\_java\_drawing.source.menus.DotsMenu.ColorDialog


createSelectionAction() - Method in class RNAstructure\_java\_drawing.source.menus.DotsMenu.RangeDialog


createSelectionAction() - Method in class RNAstructure\_java\_drawing.source.menus.StructureMenu.MoveDialog


createSelectionAction() - Method in class RNAstructure\_java\_interface.source.menus.ConstraintsMenu.NucOrHelixDialog


createSelectionAction() - Method in class RNAstructure\_java\_interface.source.windows.OligoWalkWindow.OligoSelectionDialog


createSelectionAction() - Method in class RNAstructure\_java\_utilities.ValueSelectionDialog
:   Create the action that occurs once values are selected.

currentColor - Variable in class RNAstructure\_java\_interface.source.windows.OligoWalkWindow.BarButton
:   The current color painted on this button.


## D

data - Variable in class RNAstructure\_java\_drawing.source.dialogs.StructureDialog
:   The array of data for a particular structure.

defaultColor - Variable in class RNAstructure\_java\_interface.source.windows.OligoWalkWindow.BarButton
:   The default color forthis button.

defaultFile - Variable in class RNAstructure\_java\_utilities.FileChooser
:   The default file name for this file chooser, if necessary.

delete() - Method in class RNAstructure\_java\_drawing.source.proxy.DotPlotBackend


delete() - Method in class RNAstructure\_java\_drawing.source.proxy.StructureBackend


delete() - Method in class RNAstructure\_java\_interface.source.startup.RNAstructureBackendCalculator


delete\_DotPlotBackend(long) - Static method in class RNAstructure\_java\_drawing.source.proxy.DotPlotBackendProxyJNI


delete\_RNAstructureBackendCalculator(long) - Static method in class RNAstructure\_java\_interface.source.startup.RNAstructureBackendCalculatorProxyJNI


delete\_StructureBackend(long) - Static method in class RNAstructure\_java\_drawing.source.proxy.StructureBackendProxyJNI


deleteBackendTuple(int) - Method in class RNAstructure\_java\_interface.source.windows.MultilignWindow


deleteBackendTuple(int) - Method in class RNAstructure\_java\_interface.source.windows.MultiWindow
:   Delete a tuple from the calculation back end.

deleteBackendTuple(int) - Method in class RNAstructure\_java\_interface.source.windows.TurboFoldWindow


deleteMultilignTuple(long) - Method in class RNAstructure\_java\_interface.source.startup.RNAstructureBackendCalculator


deleteTurboFoldTuple(long) - Method in class RNAstructure\_java\_interface.source.startup.RNAstructureBackendCalculator


deselect() - Method in class RNAstructure\_java\_interface.source.windows.OligoWalkWindow.BarButton
:   Deselect this button.

determineOligoMaximum(int) - Method in class RNAstructure\_java\_interface.source.startup.RNAstructureBackendCalculator


dialog - Variable in class RNAstructure\_java\_drawing.source.menus.AnnotationMenu
:   The structure panel connected to this menu.

dialog - Variable in class RNAstructure\_java\_drawing.source.menus.DotsMenu
:   The structure dialog connected to this menu.

dialog - Variable in class RNAstructure\_java\_drawing.source.menus.PlotMenu
:   The plot dialog connected to this menu.

dialog - Variable in class RNAstructure\_java\_drawing.source.menus.StructureMenu
:   The structure dialog connected to this menu.

dialog - Variable in class RNAstructure\_java\_interface.source.windows.DrawingWindow
:   The image dialog connected to this window.

dialogHandler - Variable in class RNAstructure\_java\_interface.source.windows.InternalWindow
:   A dialog handler to show any simple dialog messages that pop up from
    this window.

doActions(String) - Method in class RNAstructure\_java\_interface.source.windows.DrawingWindow
:   Do any actions specific to this window.

doActions(String) - Method in class RNAstructure\_java\_interface.source.windows.InternalWindow
:   Do any actions specific to this window.

doActions(String) - Method in class RNAstructure\_java\_interface.source.windows.ModuleWindow


doActions(String) - Method in class RNAstructure\_java\_interface.source.windows.SequenceDisplayWindow


doAdditionalCheck(char) - Method in class RNAstructure\_java\_utilities.NumberField
:   Do any additional necessary checks on a key character to make sure it's
    valid to be output.

doAdditionalCheck(char) - Method in class RNAstructure\_java\_utilities.NumberField.DoubleField


doAdditionalCheck(char) - Method in class RNAstructure\_java\_utilities.NumberField.FloatField


doMenuActions(String) - Method in class RNAstructure\_java\_drawing.source.dialogs.ImageDialog.PrintMenu


doMenuActions(String) - Method in class RNAstructure\_java\_drawing.source.menus.AnnotationMenu


doMenuActions(String) - Method in class RNAstructure\_java\_drawing.source.menus.DotsMenu


doMenuActions(String) - Method in class RNAstructure\_java\_drawing.source.menus.PlotMenu


doMenuActions(String) - Method in class RNAstructure\_java\_drawing.source.menus.StructureMenu


doMenuActions(String) - Method in class RNAstructure\_java\_interface.source.menus.ConstraintsMenu


doMenuActions(String) - Method in class RNAstructure\_java\_interface.source.menus.FileMenu


doMenuActions(String) - Method in class RNAstructure\_java\_interface.source.menus.HelpMenu


doMenuActions(String) - Method in class RNAstructure\_java\_interface.source.menus.NucleicAcidMenu


doMenuActions(String) - Method in class RNAstructure\_java\_interface.source.menus.RolloverMenu


doMenuActions(String) - Method in class RNAstructure\_java\_interface.source.windows.OligoWalkWindow.OligoWalkResultsMenu


doMenuActions(String) - Method in class RNAstructure\_java\_interface.source.windows.SequenceDisplayWindow.EditMenu


doMenuActions(String) - Method in class RNAstructure\_java\_interface.source.windows.SequenceDisplayWindow.ReadMenu


doMenuActions(String) - Method in class RNAstructure\_java\_utilities.RNAstructureMenu
:   Do any actions specific to this menu.

doOpen() - Static method in class RNAstructure\_java\_utilities.FileChooser
:   Create a file chooser that selects a file to open.

doRevalidate() - Method in class RNAstructure\_java\_interface.source.windows.DrawingWindow
:   Revalidate the drawing window.

doSave() - Static method in class RNAstructure\_java\_utilities.FileChooser
:   Create a file chooser that selects a file to save.

doSave(String) - Static method in class RNAstructure\_java\_utilities.FileChooser
:   Create a file chooser that selects a file to save, and will have a
    default file filled in.

DotPlotBackend - Class in RNAstructure\_java\_drawing.source.proxy


DotPlotBackend(long, boolean) - Constructor for class RNAstructure\_java\_drawing.source.proxy.DotPlotBackend


DotPlotBackend() - Constructor for class RNAstructure\_java\_drawing.source.proxy.DotPlotBackend


DotPlotBackend\_getBounds(long, DotPlotBackend) - Static method in class RNAstructure\_java\_drawing.source.proxy.DotPlotBackendProxyJNI


DotPlotBackend\_getColors(long, DotPlotBackend) - Static method in class RNAstructure\_java\_drawing.source.proxy.DotPlotBackendProxyJNI


DotPlotBackend\_getCurrentMax(long, DotPlotBackend) - Static method in class RNAstructure\_java\_drawing.source.proxy.DotPlotBackendProxyJNI


DotPlotBackend\_getCurrentMin(long, DotPlotBackend) - Static method in class RNAstructure\_java\_drawing.source.proxy.DotPlotBackendProxyJNI


DotPlotBackend\_getDefaultMax(long, DotPlotBackend) - Static method in class RNAstructure\_java\_drawing.source.proxy.DotPlotBackendProxyJNI


DotPlotBackend\_getDefaultMin(long, DotPlotBackend) - Static method in class RNAstructure\_java\_drawing.source.proxy.DotPlotBackendProxyJNI


DotPlotBackend\_getDotData(long, DotPlotBackend, int, int) - Static method in class RNAstructure\_java\_drawing.source.proxy.DotPlotBackendProxyJNI


DotPlotBackend\_getDotDataByLocation(long, DotPlotBackend, int, int, double) - Static method in class RNAstructure\_java\_drawing.source.proxy.DotPlotBackendProxyJNI


DotPlotBackend\_getGridLine(long, DotPlotBackend, int) - Static method in class RNAstructure\_java\_drawing.source.proxy.DotPlotBackendProxyJNI


DotPlotBackend\_getLegendData(long, DotPlotBackend) - Static method in class RNAstructure\_java\_drawing.source.proxy.DotPlotBackendProxyJNI


DotPlotBackend\_getLength(long, DotPlotBackend) - Static method in class RNAstructure\_java\_drawing.source.proxy.DotPlotBackendProxyJNI


DotPlotBackend\_readData(long, DotPlotBackend, String, int) - Static method in class RNAstructure\_java\_drawing.source.proxy.DotPlotBackendProxyJNI


DotPlotBackend\_setColors(long, DotPlotBackend, int) - Static method in class RNAstructure\_java\_drawing.source.proxy.DotPlotBackendProxyJNI


DotPlotBackend\_setRange(long, DotPlotBackend, double, double) - Static method in class RNAstructure\_java\_drawing.source.proxy.DotPlotBackendProxyJNI


DotPlotBackend\_writePostscriptFile(long, DotPlotBackend, String) - Static method in class RNAstructure\_java\_drawing.source.proxy.DotPlotBackendProxyJNI


DotPlotBackend\_writeStructuresFile(long, DotPlotBackend, String, String) - Static method in class RNAstructure\_java\_drawing.source.proxy.DotPlotBackendProxyJNI


DotPlotBackend\_writeSVGFile(long, DotPlotBackend, String) - Static method in class RNAstructure\_java\_drawing.source.proxy.DotPlotBackendProxyJNI


DotPlotBackend\_writeTextFile(long, DotPlotBackend, String) - Static method in class RNAstructure\_java\_drawing.source.proxy.DotPlotBackendProxyJNI


DotPlotBackendProxyJNI - Class in RNAstructure\_java\_drawing.source.proxy


DotPlotBackendProxyJNI() - Constructor for class RNAstructure\_java\_drawing.source.proxy.DotPlotBackendProxyJNI


DotsMenu - Class in RNAstructure\_java\_drawing.source.menus
:   A class that handles a menu which can manipulate a drawn dot plot.

DotsMenu(PlotDialog) - Constructor for class RNAstructure\_java\_drawing.source.menus.DotsMenu
:   Constructor.

DotsMenu.ColorDialog - Class in RNAstructure\_java\_drawing.source.menus
:   An inner class which creates a dialog that sets the number of colors in
    a dot plot.

DotsMenu.ColorDialog() - Constructor for class RNAstructure\_java\_drawing.source.menus.DotsMenu.ColorDialog
:   Constructor.

DotsMenu.RangeDialog - Class in RNAstructure\_java\_drawing.source.menus
:   An inner class which creates a dialog that sets the range of values in
    a dot plot.

DotsMenu.RangeDialog() - Constructor for class RNAstructure\_java\_drawing.source.menus.DotsMenu.RangeDialog
:   Constructor.

DrawingWindow - Class in RNAstructure\_java\_interface.source.windows
:   A class responsible for displaying a drawing window.

DrawingWindow(String) - Constructor for class RNAstructure\_java\_interface.source.windows.DrawingWindow
:   File Constructor.

DrawingWindow(String, int) - Constructor for class RNAstructure\_java\_interface.source.windows.DrawingWindow
:   Strand Constructor.

DynalignFoldWindow - Class in RNAstructure\_java\_interface.source.windows
:   A class responsible for initializing and running the Dynalign module, which
    calculates common structures for two strands of nucleic acids.

DynalignFoldWindow(String) - Constructor for class RNAstructure\_java\_interface.source.windows.DynalignFoldWindow
:   Constructor.

DynalignRefoldWindow - Class in RNAstructure\_java\_interface.source.windows
:   A class responsible for initializing and running the Dynalign refold
    module, which refolds a previous Dynalign calculation from a save file.

DynalignRefoldWindow() - Constructor for class RNAstructure\_java\_interface.source.windows.DynalignRefoldWindow
:   Constructor.

DynamicMenuBar - Class in RNAstructure\_java\_interface.source.menus
:   A class that creates a menu bar.

DynamicMenuBar(InternalWindow, RolloverMenu...) - Constructor for class RNAstructure\_java\_interface.source.menus.DynamicMenuBar
:   Constructor.


## E

edited - Variable in class RNAstructure\_java\_interface.source.windows.SequenceDisplayWindow
:   A boolean, true if the sequence has been edited, false if not.

Efn2Window - Class in RNAstructure\_java\_interface.source.windows
:   A class responsible for initializing and running the efn2 (Energy Function
    2) module, which calculates the folding free energy of structures.

Efn2Window(String) - Constructor for class RNAstructure\_java\_interface.source.windows.Efn2Window
:   Constructor.

enableMenus() - Method in class RNAstructure\_java\_interface.source.menus.DynamicMenuBar
:   Enable the menus on this menu bar.

error - Variable in class RNAstructure\_java\_drawing.source.dialogs.ImageDialog
:   The error message generated while building this panel, if any.

error - Variable in class RNAstructure\_java\_interface.source.windows.DrawingWindow
:   True if an error occurred building this window, false if not.

executeModuleAction(String) - Method in class RNAstructure\_java\_interface.source.windows.DynalignFoldWindow


executeModuleAction(String) - Method in class RNAstructure\_java\_interface.source.windows.DynalignRefoldWindow


executeModuleAction(String) - Method in class RNAstructure\_java\_interface.source.windows.Efn2Window


executeModuleAction(String) - Method in class RNAstructure\_java\_interface.source.windows.FoldDoubleWindow


executeModuleAction(String) - Method in class RNAstructure\_java\_interface.source.windows.FoldSingleWindow


executeModuleAction(String) - Method in class RNAstructure\_java\_interface.source.windows.FoldSuboptimalWindow


executeModuleAction(String) - Method in class RNAstructure\_java\_interface.source.windows.MaxExpectWindow


executeModuleAction(String) - Method in class RNAstructure\_java\_interface.source.windows.ModuleWindow
:   Execute an action within a particular module.

executeModuleAction(String) - Method in class RNAstructure\_java\_interface.source.windows.MultilignWindow


executeModuleAction(String) - Method in class RNAstructure\_java\_interface.source.windows.MultiWindow


executeModuleAction(String) - Method in class RNAstructure\_java\_interface.source.windows.OligoScreenWindow


executeModuleAction(String) - Method in class RNAstructure\_java\_interface.source.windows.OligoWalkWindow


executeModuleAction(String) - Method in class RNAstructure\_java\_interface.source.windows.PartitionDoubleWindow


executeModuleAction(String) - Method in class RNAstructure\_java\_interface.source.windows.PartitionSingleWindow


executeModuleAction(String) - Method in class RNAstructure\_java\_interface.source.windows.ProbKnotWindow


executeModuleAction(String) - Method in class RNAstructure\_java\_interface.source.windows.PseudoknotWindow


executeModuleAction(String) - Method in class RNAstructure\_java\_interface.source.windows.RefoldWindow


executeModuleAction(String) - Method in class RNAstructure\_java\_interface.source.windows.StochasticWindow


executeModuleAction(String) - Method in class RNAstructure\_java\_interface.source.windows.TurboFoldWindow


## F

FieldPanel - Class in RNAstructure\_java\_utilities
:   A class that creates a panel which holds aligned input fields.

FieldPanel(JTextField[]) - Constructor for class RNAstructure\_java\_utilities.FieldPanel
:   Constructor.

FieldPanel.FilePanel - Class in RNAstructure\_java\_utilities
:   An inner class that handles a panel which contains file fields.

FieldPanel.FilePanel(ActionListener, FileField...) - Constructor for class RNAstructure\_java\_utilities.FieldPanel.FilePanel
:   Constructor.

FieldPanel.NumberPanel - Class in RNAstructure\_java\_utilities
:   An inner class that handles a panel which contains numeric data fields.

FieldPanel.NumberPanel(NumberField...) - Constructor for class RNAstructure\_java\_utilities.FieldPanel.NumberPanel
:   Constructor.

fields - Variable in class RNAstructure\_java\_utilities.ValueSelectionDialog
:   The array of number fields this dialog holds.

file - Variable in class RNAstructure\_java\_drawing.source.dialogs.ImageDialog
:   The file that is drawn in some way on this panel.

FileChooser - Class in RNAstructure\_java\_utilities
:   A class that creates a file chooser so the user can select a file to open
    or save.

FileChooser(FileChooser.Types, String) - Constructor for class RNAstructure\_java\_utilities.FileChooser
:   Constructor.

FileChooser.Types - Enum in RNAstructure\_java\_utilities
:   An enum that holds the possible values for the file chooser type.

FileChooser.Types() - Constructor for enum RNAstructure\_java\_utilities.FileChooser.Types


FileField - Class in RNAstructure\_java\_utilities
:   A class that creates a text field which holds a file name.

FileField(String, boolean) - Constructor for class RNAstructure\_java\_utilities.FileField
:   Constructor.

FileMenu - Class in RNAstructure\_java\_interface.source.menus
:   A class that creates a "File" menu.

FileMenu(InternalWindow) - Constructor for class RNAstructure\_java\_interface.source.menus.FileMenu
:   Constructor.

finalize() - Method in class RNAstructure\_java\_drawing.source.proxy.DotPlotBackend


finalize() - Method in class RNAstructure\_java\_drawing.source.proxy.StructureBackend


finalize() - Method in class RNAstructure\_java\_interface.source.startup.RNAstructureBackendCalculator


finishModule(String) - Method in class RNAstructure\_java\_interface.source.windows.ModuleWindow
:   Handle finishing of a module.

flip() - Method in class RNAstructure\_java\_drawing.source.dialogs.StructureDialog
:   Flip the structure image horizontally.

flip() - Method in class RNAstructure\_java\_drawing.source.proxy.StructureBackend


FoldDoubleWindow - Class in RNAstructure\_java\_interface.source.windows
:   A class responsible for initializing and running the bifold module, which
    folds two strands of nucleic acids into their likely hybrid structure.

FoldDoubleWindow(String) - Constructor for class RNAstructure\_java\_interface.source.windows.FoldDoubleWindow
:   Constructor.

foldOligo(String, int, boolean, boolean, String) - Method in class RNAstructure\_java\_interface.source.startup.RNAstructureBackendCalculator


FoldSingleWindow - Class in RNAstructure\_java\_interface.source.windows
:   A class responsible for initializing and running the Fold module, which
    folds a strand of nucleic acids into their likely lowest free energy
    structure.

FoldSingleWindow(String) - Constructor for class RNAstructure\_java\_interface.source.windows.FoldSingleWindow
:   Constructor.

FoldSuboptimalWindow - Class in RNAstructure\_java\_interface.source.windows
:   A class responsible for initializing and running the AllSub module, which
    calculates the free energy of a varied group of suboptimal structures.

FoldSuboptimalWindow(String) - Constructor for class RNAstructure\_java\_interface.source.windows.FoldSuboptimalWindow
:   Constructor.

formatSequence() - Method in class RNAstructure\_java\_interface.source.windows.SequenceDisplayWindow
:   Format the sequence in the text area.


## G

getAlignment() - Method in class RNAstructure\_java\_utilities.FileChooser
:   Select an alignment file.

getAllOligoData(int) - Method in class RNAstructure\_java\_interface.source.startup.RNAstructureBackendCalculator


getArea(int) - Method in class RNAstructure\_java\_interface.source.windows.SequenceDisplayWindow
:   Get a text area from this window.

getBounds() - Method in class RNAstructure\_java\_drawing.source.proxy.DotPlotBackend


getBracket() - Method in class RNAstructure\_java\_utilities.FileChooser
:   Select a dot bracket file.

getColors() - Method in class RNAstructure\_java\_drawing.source.dialogs.PlotDialog
:   Get the range of colors allowed in this dialog.

getColors() - Method in class RNAstructure\_java\_drawing.source.proxy.DotPlotBackend


getConstraints() - Method in class RNAstructure\_java\_utilities.FileChooser
:   Select a constraints file.

getCPtr(DotPlotBackend) - Static method in class RNAstructure\_java\_drawing.source.proxy.DotPlotBackend


getCPtr(StructureBackend) - Static method in class RNAstructure\_java\_drawing.source.proxy.StructureBackend


getCPtr(RNAstructureBackendCalculator) - Static method in class RNAstructure\_java\_interface.source.startup.RNAstructureBackendCalculator


getCT() - Method in class RNAstructure\_java\_utilities.FileChooser
:   Select a CT file.

getCurrentBounds() - Method in class RNAstructure\_java\_drawing.source.dialogs.PlotDialog
:   Get the current bounds allowed in this dialog.

getCurrentMax() - Method in class RNAstructure\_java\_drawing.source.proxy.DotPlotBackend


getCurrentMin() - Method in class RNAstructure\_java\_drawing.source.proxy.DotPlotBackend


getDefaultBounds() - Method in class RNAstructure\_java\_drawing.source.dialogs.PlotDialog
:   Get the default bounds allowed in this dialog.

getDefaultMax() - Method in class RNAstructure\_java\_drawing.source.proxy.DotPlotBackend


getDefaultMin() - Method in class RNAstructure\_java\_drawing.source.proxy.DotPlotBackend


getDisplayedOligo(int) - Method in class RNAstructure\_java\_interface.source.startup.RNAstructureBackendCalculator


getDotData(int, int) - Method in class RNAstructure\_java\_drawing.source.proxy.DotPlotBackend


getDotDataByLocation(int, int, double) - Method in class RNAstructure\_java\_drawing.source.proxy.DotPlotBackend


getDotPlot() - Method in class RNAstructure\_java\_utilities.FileChooser
:   Select a dot plot file.

getDynalign() - Method in class RNAstructure\_java\_utilities.FileChooser
:   Select a Dynalign save file.

getDynalignAlignmentConstraints() - Method in class RNAstructure\_java\_interface.source.startup.RNAstructureBackendCalculator


getDynalignAlignmentWindowSize() - Method in class RNAstructure\_java\_interface.source.startup.RNAstructureBackendCalculator


getDynalignStructureWindowSize() - Method in class RNAstructure\_java\_interface.source.startup.RNAstructureBackendCalculator


getField(int) - Method in class RNAstructure\_java\_utilities.FieldPanel.NumberPanel
:   Get a particular input field.

getFile() - Method in class RNAstructure\_java\_drawing.source.dialogs.ImageDialog
:   Get the file drawn on this dialog.

getFile(int) - Method in class RNAstructure\_java\_utilities.FieldPanel.FilePanel
:   Get a file name from a particular input field.

getFile() - Method in class RNAstructure\_java\_utilities.FileField
:   Get the file name in the field.

getFolding() - Method in class RNAstructure\_java\_utilities.FileChooser
:   Select a folding save file.

getFoldingConstraints(int) - Method in class RNAstructure\_java\_interface.source.startup.RNAstructureBackendCalculator


getFoldWindowSize() - Method in class RNAstructure\_java\_interface.source.startup.RNAstructureBackendCalculator


getFrame() - Static method in class RNAstructure\_java\_interface.source.startup.ApplicationRootFrame
:   Get the top frame of the application.

getGraphRegionBegin() - Method in class RNAstructure\_java\_interface.source.startup.RNAstructureBackendCalculator


getGraphRegionEnd() - Method in class RNAstructure\_java\_interface.source.startup.RNAstructureBackendCalculator


getGridLine(int) - Method in class RNAstructure\_java\_drawing.source.proxy.DotPlotBackend


getHelix() - Method in class RNAstructure\_java\_utilities.FileChooser
:   Select a structure helix file.

getImage(String) - Static method in class RNAstructure\_java\_utilities.ImageGrabber
:   Create an image.

getImageData() - Method in class RNAstructure\_java\_interface.source.windows.DrawingWindow
:   Get the data from a dialog to put in this window.

getImageIcon(String) - Static method in class RNAstructure\_java\_utilities.ImageGrabber
:   Create an image icon.

getImageLabel(String) - Static method in class RNAstructure\_java\_utilities.ImageGrabber
:   Create an image label.

getInputControl(int) - Method in class RNAstructure\_java\_interface.source.windows.ModuleWindow
:   Get a particular input control from the content pane.

getInputControl(Container, int) - Method in class RNAstructure\_java\_interface.source.windows.ModuleWindow
:   Get a particular input control from a given container.

getLegendData() - Method in class RNAstructure\_java\_drawing.source.proxy.DotPlotBackend


getLength() - Method in class RNAstructure\_java\_drawing.source.proxy.DotPlotBackend


getList() - Method in class RNAstructure\_java\_utilities.FileChooser
:   Select an oligo list file.

getMaxConstraintIndex(int) - Method in class RNAstructure\_java\_interface.source.startup.RNAstructureBackendCalculator


getMaxLoop() - Method in class RNAstructure\_java\_interface.source.startup.RNAstructureBackendCalculator


getMaxPair() - Method in class RNAstructure\_java\_interface.source.startup.RNAstructureBackendCalculator


getMostRecentFrame() - Method in class RNAstructure\_java\_interface.source.startup.ApplicationRootFrame
:   Get the most recent internal frame in the application.

getMostStableOligo() - Method in class RNAstructure\_java\_interface.source.startup.RNAstructureBackendCalculator


getMultilignCT(int) - Method in class RNAstructure\_java\_interface.source.startup.RNAstructureBackendCalculator


getMultilignMaxPairs() - Method in class RNAstructure\_java\_interface.source.startup.RNAstructureBackendCalculator


getMultilignSequenceSetData() - Method in class RNAstructure\_java\_interface.source.startup.RNAstructureBackendCalculator


getNumMultilignSequences() - Method in class RNAstructure\_java\_interface.source.startup.RNAstructureBackendCalculator


getNumSequences() - Method in class RNAstructure\_java\_interface.source.windows.MultilignWindow


getNumSequences() - Method in class RNAstructure\_java\_interface.source.windows.MultiWindow
:   Get the number of sequences this window handles.

getNumSequences() - Method in class RNAstructure\_java\_interface.source.windows.TurboFoldWindow


getNumTurboFoldSequences() - Method in class RNAstructure\_java\_interface.source.startup.RNAstructureBackendCalculator


getOligoLabelData(int) - Method in class RNAstructure\_java\_interface.source.startup.RNAstructureBackendCalculator


getOligoTargetLength() - Method in class RNAstructure\_java\_interface.source.startup.RNAstructureBackendCalculator


getOligoTargetSequence() - Method in class RNAstructure\_java\_interface.source.startup.RNAstructureBackendCalculator


getOUT() - Method in class RNAstructure\_java\_utilities.FileChooser
:   Select a general OUT file.

getPartition() - Method in class RNAstructure\_java\_utilities.FileChooser
:   Select a partition function save file.

getPostscript() - Method in class RNAstructure\_java\_utilities.FileChooser
:   Select a Postscript image file.

getProgressNumber() - Method in class RNAstructure\_java\_interface.source.startup.RNAstructureBackendCalculator


getRefoldWindowSize() - Method in class RNAstructure\_java\_interface.source.startup.RNAstructureBackendCalculator


getReport() - Method in class RNAstructure\_java\_utilities.FileChooser
:   Select an oligo report file.

getScale() - Method in class RNAstructure\_java\_drawing.source.dialogs.ImageDialog
:   Get the scale at which the image is zoomed.

getSelectedIndex() - Method in class RNAstructure\_java\_utilities.RadioButtonPanel
:   Get the index of the selected button from this panel.

getSelectedItem() - Method in class RNAstructure\_java\_interface.source.windows.OligoWalkWindow.StringComboBox


getSelectedName() - Method in class RNAstructure\_java\_utilities.RadioButtonPanel
:   Get the command of the selected button from this panel.

getSequence() - Method in class RNAstructure\_java\_utilities.FileChooser
:   Select a sequence file.

getSequenceComment() - Method in class RNAstructure\_java\_interface.source.startup.RNAstructureBackendCalculator


getSequenceData() - Method in class RNAstructure\_java\_interface.source.startup.RNAstructureBackendCalculator


getSequenceExtended() - Method in class RNAstructure\_java\_utilities.FileChooser
:   Select a sequence file in its extended context, with more than its
    usual possible file types.

getSequenceSetAsString() - Method in class RNAstructure\_java\_interface.source.windows.MultilignWindow


getSequenceSetAsString() - Method in class RNAstructure\_java\_interface.source.windows.MultiWindow
:   Get a representation of the sequence set as a string.

getSequenceSetAsString() - Method in class RNAstructure\_java\_interface.source.windows.TurboFoldWindow


getSequenceTitle() - Method in class RNAstructure\_java\_interface.source.startup.RNAstructureBackendCalculator


getSHAPE() - Method in class RNAstructure\_java\_utilities.FileChooser
:   Select a SHAPE file.

getSpinner() - Method in class RNAstructure\_java\_interface.source.windows.OligoWalkWindow.SpinnerPanel
:   Get the spinner.

getStructureData(int) - Method in class RNAstructure\_java\_drawing.source.proxy.StructureBackend


getStructureType() - Method in class RNAstructure\_java\_interface.source.startup.RNAstructureBackendCalculator


getSuboptimalAbsoluteDiff() - Method in class RNAstructure\_java\_interface.source.startup.RNAstructureBackendCalculator


getSuboptimalPercentDiff() - Method in class RNAstructure\_java\_interface.source.startup.RNAstructureBackendCalculator


getSVG() - Method in class RNAstructure\_java\_utilities.FileChooser
:   Select an SVG file.

getTemperature() - Method in class RNAstructure\_java\_interface.source.startup.RNAstructureBackendCalculator


getToolBar() - Method in class RNAstructure\_java\_interface.source.startup.ApplicationRootFrame
:   Get the toolbar.

getTurboFoldCT(int) - Method in class RNAstructure\_java\_interface.source.startup.RNAstructureBackendCalculator


getTurboFoldSaveFile(int) - Method in class RNAstructure\_java\_interface.source.startup.RNAstructureBackendCalculator


getTurboFoldSequenceSetData() - Method in class RNAstructure\_java\_interface.source.startup.RNAstructureBackendCalculator


getValue() - Method in class RNAstructure\_java\_interface.source.windows.OligoWalkWindow.SpinnerPanel
:   Get the spinner value.

getValue() - Method in class RNAstructure\_java\_utilities.NumberField.DoubleField


getValue() - Method in class RNAstructure\_java\_utilities.NumberField.FloatField


getValue() - Method in class RNAstructure\_java\_utilities.NumberField
:   Get the text field's numeric value.

getValue() - Method in class RNAstructure\_java\_utilities.NumberField.IntegerField


group - Variable in class RNAstructure\_java\_utilities.RadioButtonPanel
:   The button group connected to this panel.

group2 - Variable in class RNAstructure\_java\_utilities.RadioButtonPanel
:   The button group, as a list, for ease of access to each.


## H

hasPairs - Variable in class RNAstructure\_java\_drawing.source.dialogs.StructureDialog
:   Whether the current structure has pairs.

HelpMenu - Class in RNAstructure\_java\_interface.source.menus
:   A class that creates a "Help" menu.

HelpMenu() - Constructor for class RNAstructure\_java\_interface.source.menus.HelpMenu
:   Constructor.

HTMLCheckBox - Class in RNAstructure\_java\_utilities
:   A class that creates a check box whose text is HTML.

HTMLCheckBox(String, boolean) - Constructor for class RNAstructure\_java\_utilities.HTMLCheckBox
:   Constructor.


## I

ImageDialog - Class in RNAstructure\_java\_drawing.source.dialogs
:   A printable and zoomable dialog that holds drawn images.

ImageDialog(String) - Constructor for class RNAstructure\_java\_drawing.source.dialogs.ImageDialog
:   Constructor.

ImageDialog.PrintMenu - Class in RNAstructure\_java\_drawing.source.dialogs
:   An inner class which creates a print menu.

ImageDialog.PrintMenu() - Constructor for class RNAstructure\_java\_drawing.source.dialogs.ImageDialog.PrintMenu
:   Constructor.

ImageDialog.ZoomDialog - Class in RNAstructure\_java\_drawing.source.dialogs
:   An inner class which creates a dialog that zooms a structure.

ImageDialog.ZoomDialog() - Constructor for class RNAstructure\_java\_drawing.source.dialogs.ImageDialog.ZoomDialog
:   Constructor.

imageDialogs - Variable in class RNAstructure\_java\_interface.source.windows.MultiWindow
:   The list of drawing windows shown after the calculation is done.

ImageGrabber - Class in RNAstructure\_java\_utilities
:   A class that creates images in various contexts for the RNAstructure GUI.

ImageGrabber() - Constructor for class RNAstructure\_java\_utilities.ImageGrabber


initial - Variable in class RNAstructure\_java\_utilities.NumberField
:   The initial default value of the field.

insertMenuItem(String, String, ActionListener, int) - Method in class RNAstructure\_java\_utilities.RNAstructureMenu
:   Insert a plain text menu item with rollover text.

InternalWindow - Class in RNAstructure\_java\_interface.source.windows
:   A class that holds internal frames for the RNAstructure GUI.

InternalWindow() - Constructor for class RNAstructure\_java\_interface.source.windows.InternalWindow
:   Constructor.

isError() - Method in class RNAstructure\_java\_drawing.source.dialogs.ImageDialog
:   Get whether this panel ran into an error while being built.

isError() - Method in class RNAstructure\_java\_interface.source.windows.DrawingWindow
:   Get whether this window encountered an error.

isError() - Method in class RNAstructure\_java\_utilities.FieldPanel.FilePanel
:   Get whether any fields are empty; an empty field is an error.

isInputWindow - Variable in class RNAstructure\_java\_interface.source.windows.OligoWalkWindow
:   A boolean that tells whether this window is an input window or not.

isMac() - Static method in class RNAstructure\_java\_utilities.MacChecker
:   Get whether the current OS is a Mac.

isRNA - Variable in class RNAstructure\_java\_interface.source.windows.ModuleWindow
:   A boolean, true if the calculation handles RNA, false if not.


## J

job - Variable in class RNAstructure\_java\_drawing.source.dialogs.ImageDialog
:   The job that prints out this panel


## L

label - Variable in class RNAstructure\_java\_drawing.source.dialogs.ImageDialog
:   The main label that describes the panel.

layout - Variable in class RNAstructure\_java\_interface.source.windows.ModuleWindow
:   The layout for this window.

legendGrid - Variable in class RNAstructure\_java\_drawing.source.dialogs.ImageDialog
:   The panel that contains the legend grid.


## M

MacChecker - Class in RNAstructure\_java\_utilities
:   A class that handles checking whether an OS is a Mac, and setting general
    properties if that is the case.

MacChecker() - Constructor for class RNAstructure\_java\_utilities.MacChecker


main(String[]) - Static method in class RNAstructure\_java\_drawing.source.runners.PlotRunner
:   The main method.

main(String[]) - Static method in class RNAstructure\_java\_drawing.source.runners.StructureRunner
:   The main method.

main(String[]) - Static method in class RNAstructure\_java\_interface.source.startup.RNAstructure
:   The main method.

mainPanel - Variable in class RNAstructure\_java\_drawing.source.dialogs.ImageDialog
:   The main panel that the drawing resides on.

makeBottomBorder(int, JComponent) - Method in class RNAstructure\_java\_utilities.BorderBuilder
:   Create an empty bottom border on a component.

makeEmptyBorder(String, int, JComponent) - Method in class RNAstructure\_java\_utilities.BorderBuilder
:   Create an empty border on a component.

makeEqualBorder(int, JComponent) - Method in class RNAstructure\_java\_utilities.BorderBuilder
:   Create an empty equidistant border on a component.

makeErrorDialog(String) - Method in class RNAstructure\_java\_utilities.SimpleDialogHandler
:   Show an error dialog.

makeHorizontal(String[]) - Static method in class RNAstructure\_java\_utilities.RadioButtonPanel
:   Create a button panel with a horizontal layout.

makeHorizontal(String, String...) - Static method in class RNAstructure\_java\_utilities.RadioButtonPanel
:   Create a button panel with a title and horizontal layout.

makeHorizontal(String, ActionListener, String...) - Static method in class RNAstructure\_java\_utilities.RadioButtonPanel
:   Create a button panel with a title, horizontal layout, and an action
    listener associated with the buttons.

makeInputControls() - Method in class RNAstructure\_java\_interface.source.windows.DynalignFoldWindow


makeInputControls() - Method in class RNAstructure\_java\_interface.source.windows.DynalignRefoldWindow


makeInputControls() - Method in class RNAstructure\_java\_interface.source.windows.Efn2Window


makeInputControls() - Method in class RNAstructure\_java\_interface.source.windows.FoldDoubleWindow


makeInputControls() - Method in class RNAstructure\_java\_interface.source.windows.FoldSingleWindow


makeInputControls() - Method in class RNAstructure\_java\_interface.source.windows.FoldSuboptimalWindow


makeInputControls() - Method in class RNAstructure\_java\_interface.source.windows.MaxExpectWindow


makeInputControls() - Method in class RNAstructure\_java\_interface.source.windows.ModuleWindow
:   Make any input controls necessary to get data from this window for a
    calculation.

makeInputControls() - Method in class RNAstructure\_java\_interface.source.windows.MultiWindow


makeInputControls() - Method in class RNAstructure\_java\_interface.source.windows.OligoScreenWindow


makeInputControls() - Method in class RNAstructure\_java\_interface.source.windows.OligoWalkWindow
:   Make any input controls necessary to get data from this window for a
    calculation.

makeInputControls() - Method in class RNAstructure\_java\_interface.source.windows.PartitionDoubleWindow


makeInputControls() - Method in class RNAstructure\_java\_interface.source.windows.PartitionSingleWindow


makeInputControls() - Method in class RNAstructure\_java\_interface.source.windows.ProbKnotWindow


makeInputControls() - Method in class RNAstructure\_java\_interface.source.windows.PseudoknotWindow


makeInputControls() - Method in class RNAstructure\_java\_interface.source.windows.RefoldWindow


makeInputControls() - Method in class RNAstructure\_java\_interface.source.windows.StochasticWindow


makeInputDialog(NumberField.DoubleField) - Method in class RNAstructure\_java\_utilities.SimpleDialogHandler
:   Show an input dialog that gets a double within a certain range.

makeInputDialog(NumberField.IntegerField) - Method in class RNAstructure\_java\_utilities.SimpleDialogHandler
:   Show an input dialog that gets an integer within a certain range.

makeLeftBorder(int, JComponent) - Method in class RNAstructure\_java\_utilities.BorderBuilder
:   Create an empty left border on a component.

makeMessageDialog(Object) - Method in class RNAstructure\_java\_utilities.SimpleDialogHandler
:   Show a message dialog.

makeMessageDialog(String) - Method in class RNAstructure\_java\_utilities.SimpleDialogHandler
:   Show a message dialog.

makePanel() - Method in class RNAstructure\_java\_utilities.FieldPanel
:   Build the panel.

makeRightBorder(int, JComponent) - Method in class RNAstructure\_java\_utilities.BorderBuilder
:   Create an empty right border on a component.

makeStartButton(int, int) - Method in class RNAstructure\_java\_interface.source.windows.ModuleWindow
:   Create the "START" button.

makeThreeChoicesDialog(Object) - Method in class RNAstructure\_java\_utilities.SimpleDialogHandler
:   Show a dialog that gives the user one of three choices to make.

makeThreeChoicesDialog(String) - Method in class RNAstructure\_java\_utilities.SimpleDialogHandler
:   Show a dialog that gives the user one of three choices to make.

makeTitledBorder(String, JComponent) - Method in class RNAstructure\_java\_utilities.BorderBuilder
:   Create a padded titled border on a component.

makeTopBorder(int, JComponent) - Method in class RNAstructure\_java\_utilities.BorderBuilder
:   Create an empty top border on a component.

makeTwoChoicesDialog(Object) - Method in class RNAstructure\_java\_utilities.SimpleDialogHandler
:   Show a dialog that gives the user one of two choices to make.

makeTwoChoicesDialog(String) - Method in class RNAstructure\_java\_utilities.SimpleDialogHandler
:   Show a dialog that gives the user one of two choices to make.

makeVertical(String[]) - Static method in class RNAstructure\_java\_utilities.RadioButtonPanel
:   Create a button panel with a vertical layout.

makeVertical(String, String...) - Static method in class RNAstructure\_java\_utilities.RadioButtonPanel
:   Create a button panel with a title and vertical layout.

makeVertical(String, ActionListener, String...) - Static method in class RNAstructure\_java\_utilities.RadioButtonPanel
:   Create a button panel with a title, vertical layout, and an action
    listener associated with the buttons.

makeWarningDialog(String) - Method in class RNAstructure\_java\_utilities.SimpleDialogHandler
:   Show a warning dialog.

maxBoundX - Variable in class RNAstructure\_java\_drawing.source.dialogs.ImageDialog
:   The maximum X bound of the image.

maxBoundY - Variable in class RNAstructure\_java\_drawing.source.dialogs.ImageDialog
:   The maximum Y bound of the image.

MaxExpectWindow - Class in RNAstructure\_java\_interface.source.windows
:   A class responsible for initializing and running the MaxExpect module,
    which generates a group of structures containing pairs with the maximum
    probability of being accurate.

MaxExpectWindow(String) - Constructor for class RNAstructure\_java\_interface.source.windows.MaxExpectWindow
:   Constructor.

maximum - Variable in class RNAstructure\_java\_utilities.NumberField
:   The maximum value of the field.

menuBar - Variable in class RNAstructure\_java\_interface.source.windows.InternalWindow
:   The menu bar that's attached to this window.

minimum - Variable in class RNAstructure\_java\_utilities.NumberField
:   The minimum value of the field.

modeNames - Static variable in class RNAstructure\_java\_interface.source.windows.TurboFoldWindow
:   An array that holds the names of the TurboFold modes.

moduleInitialized(String) - Method in class RNAstructure\_java\_interface.source.windows.ModuleWindow
:   Check to see if the module was initialized correctly.

ModuleWindow - Class in RNAstructure\_java\_interface.source.windows
:   A class responsible for creating and displaying a window that enables a
    user to input data for one of the RNAstructure modules.

ModuleWindow(String, String) - Constructor for class RNAstructure\_java\_interface.source.windows.ModuleWindow
:   Constructor.

mouseClicked(MouseEvent) - Method in class RNAstructure\_java\_interface.source.menus.RolloverMenu.RolloverListener


mouseEntered(MouseEvent) - Method in class RNAstructure\_java\_interface.source.menus.RolloverMenu.RolloverListener


mouseExited(MouseEvent) - Method in class RNAstructure\_java\_interface.source.menus.RolloverMenu.RolloverListener


mousePressed(MouseEvent) - Method in class RNAstructure\_java\_interface.source.menus.RolloverMenu.RolloverListener


mouseReleased(MouseEvent) - Method in class RNAstructure\_java\_interface.source.menus.RolloverMenu.RolloverListener


MultilignWindow - Class in RNAstructure\_java\_interface.source.windows
:   A class responsible for initializing and running the Multilign module,
    which calculates common secondary structures of multiple sequences using
    multiple iterations of Dynalign.

MultilignWindow(String) - Constructor for class RNAstructure\_java\_interface.source.windows.MultilignWindow
:   Constructor.

MultiWindow - Class in RNAstructure\_java\_interface.source.windows
:   A class responsible for creating and displaying an input window that takes
    multiple arbitrary sequences and CT files as input.

MultiWindow(String, String) - Constructor for class RNAstructure\_java\_interface.source.windows.MultiWindow
:   Constructor.


## N

names - Variable in class RNAstructure\_java\_utilities.RadioButtonPanel
:   The names of the buttons in the group.

NEVER - Static variable in class RNAstructure\_java\_utilities.ScrollerPane
:   A generic constant symbolizing that a scroll bar must never be used.

new\_DotPlotBackend() - Static method in class RNAstructure\_java\_drawing.source.proxy.DotPlotBackendProxyJNI


new\_RNAstructureBackendCalculator() - Static method in class RNAstructure\_java\_interface.source.startup.RNAstructureBackendCalculatorProxyJNI


new\_StructureBackend() - Static method in class RNAstructure\_java\_drawing.source.proxy.StructureBackendProxyJNI


notMac() - Static method in class RNAstructure\_java\_utilities.MacChecker
:   Get whether the current OS is not a Mac.

NucleicAcidMenu - Class in RNAstructure\_java\_interface.source.menus
:   A class that creates a menu which holds commands specific for a nucleic
    acid type.

NucleicAcidMenu(String) - Constructor for class RNAstructure\_java\_interface.source.menus.NucleicAcidMenu
:   Constructor.

number - Variable in class RNAstructure\_java\_drawing.source.dialogs.StructureDialog
:   The structure number viewed on this panel.

NumberField - Class in RNAstructure\_java\_utilities
:   A class that creates a text field whose contents may be bounded by a
    minimum or maximum value.

NumberField(String, Number, Number, Number) - Constructor for class RNAstructure\_java\_utilities.NumberField
:   Constructor.

NumberField.DoubleField - Class in RNAstructure\_java\_utilities
:   An inner class that handles a field which contains a double value.

NumberField.DoubleField(String, Number) - Constructor for class RNAstructure\_java\_utilities.NumberField.DoubleField
:   Unbounded Constructor.

NumberField.DoubleField(String, Number, Number) - Constructor for class RNAstructure\_java\_utilities.NumberField.DoubleField
:   Minimum Bounds Constructor.

NumberField.DoubleField(String, Number, Number, Number) - Constructor for class RNAstructure\_java\_utilities.NumberField.DoubleField
:   Minimum and Maximum Bounds Constructor.

NumberField.FloatField - Class in RNAstructure\_java\_utilities
:   An inner class that handles a field which contains a float value.

NumberField.FloatField(String, Number) - Constructor for class RNAstructure\_java\_utilities.NumberField.FloatField
:   Unbounded Constructor.

NumberField.FloatField(String, Number, Number) - Constructor for class RNAstructure\_java\_utilities.NumberField.FloatField
:   Minimum Bounds Constructor.

NumberField.FloatField(String, Number, Number, Number) - Constructor for class RNAstructure\_java\_utilities.NumberField.FloatField
:   Minimum and Maximum Bounds Constructor.

NumberField.IntegerField - Class in RNAstructure\_java\_utilities
:   An inner class that handles a field which contains an integer value.

NumberField.IntegerField(String, Integer) - Constructor for class RNAstructure\_java\_utilities.NumberField.IntegerField
:   Unbounded Constructor.

NumberField.IntegerField(String, Integer, Integer) - Constructor for class RNAstructure\_java\_utilities.NumberField.IntegerField
:   Minimum Bounds Constructor.

NumberField.IntegerField(String, Integer, Integer, Integer) - Constructor for class RNAstructure\_java\_utilities.NumberField.IntegerField
:   Minimum and Maximum Bounds Constructor.


## O

oligomerChemistry - Static variable in class RNAstructure\_java\_interface.source.windows.OligoScreenWindow
:   The oligomer chemistry.

OligoScreenWindow - Class in RNAstructure\_java\_interface.source.windows
:   A class responsible for initializing and running the OligoScreen module,
    which calculates thermodynamic properties for a list of oligonucleotides.

OligoScreenWindow() - Constructor for class RNAstructure\_java\_interface.source.windows.OligoScreenWindow
:   Constructor.

OligoWalkWindow - Class in RNAstructure\_java\_interface.source.windows
:   A class that handles creation of windows for the OligoWalk module.

OligoWalkWindow() - Constructor for class RNAstructure\_java\_interface.source.windows.OligoWalkWindow
:   Constructor.

OligoWalkWindow.BarButton - Class in RNAstructure\_java\_interface.source.windows
:   An inner class that encapsulates a bar button.

OligoWalkWindow.BarButton() - Constructor for class RNAstructure\_java\_interface.source.windows.OligoWalkWindow.BarButton
:   Constructor.

OligoWalkWindow.OligoSelectionDialog - Class in RNAstructure\_java\_interface.source.windows
:   An inner class which creates a dialog that selects a particular oligo
    to move to in the results window.

OligoWalkWindow.OligoSelectionDialog() - Constructor for class RNAstructure\_java\_interface.source.windows.OligoWalkWindow.OligoSelectionDialog
:   Constructor.

OligoWalkWindow.OligoWalkResultsMenu - Class in RNAstructure\_java\_interface.source.windows
:   An inner class that handles creation of the oligo graph menu for the
    OligoWalk results window.

OligoWalkWindow.OligoWalkResultsMenu() - Constructor for class RNAstructure\_java\_interface.source.windows.OligoWalkWindow.OligoWalkResultsMenu
:   Constructor.

OligoWalkWindow.SpinnerPanel - Class in RNAstructure\_java\_interface.source.windows
:   An class that creates a panel containing a single spinner with a label.

OligoWalkWindow.SpinnerPanel(String, int, int, int) - Constructor for class RNAstructure\_java\_interface.source.windows.OligoWalkWindow.SpinnerPanel
:   Constructor.

OligoWalkWindow.StringComboBox - Class in RNAstructure\_java\_interface.source.windows
:   An inner class that creates a combo box holding a string list.

OligoWalkWindow.StringComboBox(String...) - Constructor for class RNAstructure\_java\_interface.source.windows.OligoWalkWindow.StringComboBox
:   Constructor.

options - Variable in class RNAstructure\_java\_interface.source.windows.MultiWindow
:   The box that holds a particular window's unique options.


## P

paintComponent(Graphics) - Method in class RNAstructure\_java\_interface.source.windows.OligoWalkWindow.BarButton


panelInput - Variable in class RNAstructure\_java\_interface.source.windows.OligoWalkWindow


panelResults - Variable in class RNAstructure\_java\_interface.source.windows.OligoWalkWindow
:   The box that holds the results.

PartitionDoubleWindow - Class in RNAstructure\_java\_interface.source.windows
:   A class responsible for initializing and running the bipartition module,
    which calculates the partition function for two strands of nucleic acids.

PartitionDoubleWindow(String) - Constructor for class RNAstructure\_java\_interface.source.windows.PartitionDoubleWindow
:   Constructor.

PartitionSingleWindow - Class in RNAstructure\_java\_interface.source.windows
:   A class responsible for initializing and running the partition module, which
    calculates the partition function for a single strand of nucleic acids.

PartitionSingleWindow(String) - Constructor for class RNAstructure\_java\_interface.source.windows.PartitionSingleWindow
:   Constructor.

placeComponent(int, int, JComponent) - Method in class RNAstructure\_java\_interface.source.windows.ModuleWindow
:   .

playClip(char) - Method in class RNAstructure\_java\_interface.source.windows.SequenceDisplayWindow
:   Play a sound clip, depending on the nucleotide.

PlotDialog - Class in RNAstructure\_java\_drawing.source.dialogs
:   A dialog that holds dot plot images.

PlotDialog(String) - Constructor for class RNAstructure\_java\_drawing.source.dialogs.PlotDialog
:   File Constructor.

PlotDialog(String, int) - Constructor for class RNAstructure\_java\_drawing.source.dialogs.PlotDialog
:   Strand Constructor.

plotLength - Variable in class RNAstructure\_java\_drawing.source.dialogs.PlotDialog
:   The length of the plot.

PlotMenu - Class in RNAstructure\_java\_drawing.source.menus
:   A class that handles a menu which can manipulate a drawn dot plot.

PlotMenu(PlotDialog) - Constructor for class RNAstructure\_java\_drawing.source.menus.PlotMenu
:   Constructor.

PlotRunner - Class in RNAstructure\_java\_drawing.source.runners
:   A class that runs RNAstructure Java dot plot drawing independently of the
    main RNAstructure application.

PlotRunner() - Constructor for class RNAstructure\_java\_drawing.source.runners.PlotRunner
:   Constructor.

position - Variable in class RNAstructure\_java\_interface.source.windows.SequenceDisplayWindow
:   The position at which reading is occurring.

preferredDialogWidth - Variable in class RNAstructure\_java\_drawing.source.dialogs.ImageDialog
:   The preferred dialog width.

print(Graphics, PageFormat, int) - Method in class RNAstructure\_java\_drawing.source.dialogs.ImageDialog
:   Render the panel for the print job.

printPanel() - Method in class RNAstructure\_java\_drawing.source.dialogs.ImageDialog
:   Print if the user decides to, then reset the printer job properties.

ProbKnotWindow - Class in RNAstructure\_java\_interface.source.windows
:   A class responsible for initializing and running the ProbKnot module, which
    predicts pseudoknots in a strand of nucleic acids.

ProbKnotWindow(String) - Constructor for class RNAstructure\_java\_interface.source.windows.ProbKnotWindow
:   Constructor.

progressBar - Variable in class RNAstructure\_java\_interface.source.windows.ModuleWindow
:   The progress bar dialog for this window.

PseudoknotWindow - Class in RNAstructure\_java\_interface.source.windows
:   A class responsible for initializing and running the RemovePseudoknots
    module, which removes pseudoknots from a nucleic acid sequence.

PseudoknotWindow(String) - Constructor for class RNAstructure\_java\_interface.source.windows.PseudoknotWindow
:   Constructor.


## R

RadioButtonPanel - Class in RNAstructure\_java\_utilities
:   A class that creates a panel containing a group of radio buttons.

RadioButtonPanel(String, String, ActionListener, String...) - Constructor for class RNAstructure\_java\_utilities.RadioButtonPanel
:   Constructor.

range - Variable in class RNAstructure\_java\_drawing.source.menus.DotsMenu.RangeDialog
:   The number panel that holds the range data.

readButton - Variable in class RNAstructure\_java\_interface.source.windows.SequenceDisplayWindow
:   The button which can be clicked to start or stop reading.

readData(String, int) - Method in class RNAstructure\_java\_drawing.source.proxy.DotPlotBackend


readDynalignAlignmentConstraintsFile(String) - Method in class RNAstructure\_java\_interface.source.startup.RNAstructureBackendCalculator


readFoldingConstraintsFile(String, int) - Method in class RNAstructure\_java\_interface.source.startup.RNAstructureBackendCalculator


readImageData() - Method in class RNAstructure\_java\_drawing.source.dialogs.ImageDialog
:   Read image data from a particular file.

readImageData() - Method in class RNAstructure\_java\_drawing.source.dialogs.PlotDialog


readImageData() - Method in class RNAstructure\_java\_drawing.source.dialogs.StructureDialog


reading - Variable in class RNAstructure\_java\_interface.source.windows.SequenceDisplayWindow
:   A boolean, true if reading while typing is active, false if not.

readSequenceData(String) - Method in class RNAstructure\_java\_interface.source.startup.RNAstructureBackendCalculator


readStructureData(String) - Method in class RNAstructure\_java\_drawing.source.proxy.StructureBackend


RefoldWindow - Class in RNAstructure\_java\_interface.source.windows
:   A class responsible for initializing and running the refold module, which
    refolds a previous folding calculation from a save file.

RefoldWindow() - Constructor for class RNAstructure\_java\_interface.source.windows.RefoldWindow
:   Constructor.

refresh() - Method in class RNAstructure\_java\_drawing.source.dialogs.ImageDialog
:   Refresh this dialog.

regex - Variable in class RNAstructure\_java\_drawing.source.dialogs.StructureDialog
:   The array of regexes used to pull data about a structure.

removeAnnotation() - Method in class RNAstructure\_java\_drawing.source.proxy.StructureBackend


replaceExtension(String, String) - Method in class RNAstructure\_java\_interface.source.windows.ModuleWindow
:   Replace the extension on a file name.

reset() - Method in class RNAstructure\_java\_interface.source.menus.RolloverMenu.RolloverListener
:   Reset the main frame message bar text to its default.

resetField(Number) - Method in class RNAstructure\_java\_utilities.NumberField
:   Reset a field value, if it's within bounds.

resetField(Number, Number, Number) - Method in class RNAstructure\_java\_utilities.NumberField
:   Reset a field value and bounds.

resetFrame() - Method in class RNAstructure\_java\_interface.source.startup.ApplicationRootFrame
:   Reset the frame to its original state before any windows were opened.

resetInfoLabel() - Method in class RNAstructure\_java\_interface.source.startup.ApplicationRootFrame
:   Reset the info bar at the bottom of the main window to its default.

resetModel(int, int, int) - Method in class RNAstructure\_java\_interface.source.windows.OligoWalkWindow.SpinnerPanel
:   Reset the model for the spinner.

RNAstructure - Class in RNAstructure\_java\_interface.source.startup
:   A class that starts the RNAstructure interface.

RNAstructure() - Constructor for class RNAstructure\_java\_interface.source.startup.RNAstructure


RNAstructure\_java\_drawing.source - package RNAstructure\_java\_drawing.source
:   A package that encapsulates all the Java classes that handle structure and dot plot drawing.

RNAstructure\_java\_drawing.source.dialogs - package RNAstructure\_java\_drawing.source.dialogs
:   A package that holds all Java drawing dialogs.

RNAstructure\_java\_drawing.source.menus - package RNAstructure\_java\_drawing.source.menus
:   A package that holds all menus used in the drawing framework.

RNAstructure\_java\_drawing.source.proxy - package RNAstructure\_java\_drawing.source.proxy
:   A package that holds all Java drawing classes generated by SWIG that connect the C++ back end to the Java front end.

RNAstructure\_java\_drawing.source.runners - package RNAstructure\_java\_drawing.source.runners
:   A package that holds the run templates that are used when the Java drawing framework is wrapped into self-contained executables.

RNAstructure\_java\_interface.source - package RNAstructure\_java\_interface.source
:   A package that encapsulates all the Java classes that comprise the RNAstructure Java interface.

RNAstructure\_java\_interface.source.menus - package RNAstructure\_java\_interface.source.menus
:   A package that holds all menus used in the Java RNAstructure interface.

RNAstructure\_java\_interface.source.startup - package RNAstructure\_java\_interface.source.startup
:   A package that holds all Java RNAstructure interface classes that are directly called at the Java interface startup.

RNAstructure\_java\_interface.source.windows - package RNAstructure\_java\_interface.source.windows
:   A package that holds all internal windows used in the drawing framework.

RNAstructure\_java\_utilities - package RNAstructure\_java\_utilities
:   A package that holds a variety of utilities classes.

RNAstructureBackendCalculator - Class in RNAstructure\_java\_interface.source.startup


RNAstructureBackendCalculator(long, boolean) - Constructor for class RNAstructure\_java\_interface.source.startup.RNAstructureBackendCalculator


RNAstructureBackendCalculator() - Constructor for class RNAstructure\_java\_interface.source.startup.RNAstructureBackendCalculator


RNAstructureBackendCalculator\_activateMultilign(long, RNAstructureBackendCalculator) - Static method in class RNAstructure\_java\_interface.source.startup.RNAstructureBackendCalculatorProxyJNI


RNAstructureBackendCalculator\_activateTurboFold(long, RNAstructureBackendCalculator) - Static method in class RNAstructure\_java\_interface.source.startup.RNAstructureBackendCalculatorProxyJNI


RNAstructureBackendCalculator\_addMultilignTuple(long, RNAstructureBackendCalculator, String, String) - Static method in class RNAstructure\_java\_interface.source.startup.RNAstructureBackendCalculatorProxyJNI


RNAstructureBackendCalculator\_addTurboFoldTuple(long, RNAstructureBackendCalculator, String, String) - Static method in class RNAstructure\_java\_interface.source.startup.RNAstructureBackendCalculatorProxyJNI


RNAstructureBackendCalculator\_buildAllSubDataStructure(long, RNAstructureBackendCalculator, String, boolean) - Static method in class RNAstructure\_java\_interface.source.startup.RNAstructureBackendCalculatorProxyJNI


RNAstructureBackendCalculator\_buildBifoldDataStructure(long, RNAstructureBackendCalculator, String, String, boolean) - Static method in class RNAstructure\_java\_interface.source.startup.RNAstructureBackendCalculatorProxyJNI


RNAstructureBackendCalculator\_buildBipartitionDataStructure(long, RNAstructureBackendCalculator, String, String, boolean) - Static method in class RNAstructure\_java\_interface.source.startup.RNAstructureBackendCalculatorProxyJNI


RNAstructureBackendCalculator\_buildDynalignDataStructure(long, RNAstructureBackendCalculator, String, String, boolean) - Static method in class RNAstructure\_java\_interface.source.startup.RNAstructureBackendCalculatorProxyJNI


RNAstructureBackendCalculator\_buildEfn2DataStructure(long, RNAstructureBackendCalculator, String, boolean) - Static method in class RNAstructure\_java\_interface.source.startup.RNAstructureBackendCalculatorProxyJNI


RNAstructureBackendCalculator\_buildFoldDataStructure(long, RNAstructureBackendCalculator, String, boolean) - Static method in class RNAstructure\_java\_interface.source.startup.RNAstructureBackendCalculatorProxyJNI


RNAstructureBackendCalculator\_buildMaxExpectDataStructure(long, RNAstructureBackendCalculator, String) - Static method in class RNAstructure\_java\_interface.source.startup.RNAstructureBackendCalculatorProxyJNI


RNAstructureBackendCalculator\_buildOligoScreenDataStructure(long, RNAstructureBackendCalculator) - Static method in class RNAstructure\_java\_interface.source.startup.RNAstructureBackendCalculatorProxyJNI


RNAstructureBackendCalculator\_buildOligoWalkDataStructure(long, RNAstructureBackendCalculator, String, boolean) - Static method in class RNAstructure\_java\_interface.source.startup.RNAstructureBackendCalculatorProxyJNI


RNAstructureBackendCalculator\_buildPartitionDataStructure(long, RNAstructureBackendCalculator, String, boolean) - Static method in class RNAstructure\_java\_interface.source.startup.RNAstructureBackendCalculatorProxyJNI


RNAstructureBackendCalculator\_buildProbKnotDataStructure(long, RNAstructureBackendCalculator, String) - Static method in class RNAstructure\_java\_interface.source.startup.RNAstructureBackendCalculatorProxyJNI


RNAstructureBackendCalculator\_buildRefoldDynalignDataStructure(long, RNAstructureBackendCalculator, String) - Static method in class RNAstructure\_java\_interface.source.startup.RNAstructureBackendCalculatorProxyJNI


RNAstructureBackendCalculator\_buildRefoldSingleDataStructure(long, RNAstructureBackendCalculator, String) - Static method in class RNAstructure\_java\_interface.source.startup.RNAstructureBackendCalculatorProxyJNI


RNAstructureBackendCalculator\_buildRemovePseudoknotsDataStructure(long, RNAstructureBackendCalculator, String, boolean) - Static method in class RNAstructure\_java\_interface.source.startup.RNAstructureBackendCalculatorProxyJNI


RNAstructureBackendCalculator\_buildStochasticDataStructure(long, RNAstructureBackendCalculator, String) - Static method in class RNAstructure\_java\_interface.source.startup.RNAstructureBackendCalculatorProxyJNI


RNAstructureBackendCalculator\_canFoldOligoOligo(long, RNAstructureBackendCalculator, int) - Static method in class RNAstructure\_java\_interface.source.startup.RNAstructureBackendCalculatorProxyJNI


RNAstructureBackendCalculator\_canFoldOligoSelf(long, RNAstructureBackendCalculator, int) - Static method in class RNAstructure\_java\_interface.source.startup.RNAstructureBackendCalculatorProxyJNI


RNAstructureBackendCalculator\_clearDynalignAlignmentConstraints(long, RNAstructureBackendCalculator) - Static method in class RNAstructure\_java\_interface.source.startup.RNAstructureBackendCalculatorProxyJNI


RNAstructureBackendCalculator\_clearFoldingConstraints(long, RNAstructureBackendCalculator, int) - Static method in class RNAstructure\_java\_interface.source.startup.RNAstructureBackendCalculatorProxyJNI


RNAstructureBackendCalculator\_deleteMultilignTuple(long, RNAstructureBackendCalculator, long) - Static method in class RNAstructure\_java\_interface.source.startup.RNAstructureBackendCalculatorProxyJNI


RNAstructureBackendCalculator\_deleteTurboFoldTuple(long, RNAstructureBackendCalculator, long) - Static method in class RNAstructure\_java\_interface.source.startup.RNAstructureBackendCalculatorProxyJNI


RNAstructureBackendCalculator\_determineOligoMaximum(long, RNAstructureBackendCalculator, int) - Static method in class RNAstructure\_java\_interface.source.startup.RNAstructureBackendCalculatorProxyJNI


RNAstructureBackendCalculator\_foldOligo(long, RNAstructureBackendCalculator, String, int, boolean, boolean, String) - Static method in class RNAstructure\_java\_interface.source.startup.RNAstructureBackendCalculatorProxyJNI


RNAstructureBackendCalculator\_getAllOligoData(long, RNAstructureBackendCalculator, int) - Static method in class RNAstructure\_java\_interface.source.startup.RNAstructureBackendCalculatorProxyJNI


RNAstructureBackendCalculator\_getDisplayedOligo(long, RNAstructureBackendCalculator, int) - Static method in class RNAstructure\_java\_interface.source.startup.RNAstructureBackendCalculatorProxyJNI


RNAstructureBackendCalculator\_getDynalignAlignmentConstraints(long, RNAstructureBackendCalculator) - Static method in class RNAstructure\_java\_interface.source.startup.RNAstructureBackendCalculatorProxyJNI


RNAstructureBackendCalculator\_getDynalignAlignmentWindowSize(long, RNAstructureBackendCalculator) - Static method in class RNAstructure\_java\_interface.source.startup.RNAstructureBackendCalculatorProxyJNI


RNAstructureBackendCalculator\_getDynalignStructureWindowSize(long, RNAstructureBackendCalculator) - Static method in class RNAstructure\_java\_interface.source.startup.RNAstructureBackendCalculatorProxyJNI


RNAstructureBackendCalculator\_getFoldingConstraints(long, RNAstructureBackendCalculator, int) - Static method in class RNAstructure\_java\_interface.source.startup.RNAstructureBackendCalculatorProxyJNI


RNAstructureBackendCalculator\_getFoldWindowSize(long, RNAstructureBackendCalculator) - Static method in class RNAstructure\_java\_interface.source.startup.RNAstructureBackendCalculatorProxyJNI


RNAstructureBackendCalculator\_getGraphRegionBegin(long, RNAstructureBackendCalculator) - Static method in class RNAstructure\_java\_interface.source.startup.RNAstructureBackendCalculatorProxyJNI


RNAstructureBackendCalculator\_getGraphRegionEnd(long, RNAstructureBackendCalculator) - Static method in class RNAstructure\_java\_interface.source.startup.RNAstructureBackendCalculatorProxyJNI


RNAstructureBackendCalculator\_getMaxConstraintIndex(long, RNAstructureBackendCalculator, int) - Static method in class RNAstructure\_java\_interface.source.startup.RNAstructureBackendCalculatorProxyJNI


RNAstructureBackendCalculator\_getMaxLoop(long, RNAstructureBackendCalculator) - Static method in class RNAstructure\_java\_interface.source.startup.RNAstructureBackendCalculatorProxyJNI


RNAstructureBackendCalculator\_getMaxPair(long, RNAstructureBackendCalculator) - Static method in class RNAstructure\_java\_interface.source.startup.RNAstructureBackendCalculatorProxyJNI


RNAstructureBackendCalculator\_getMostStableOligo(long, RNAstructureBackendCalculator) - Static method in class RNAstructure\_java\_interface.source.startup.RNAstructureBackendCalculatorProxyJNI


RNAstructureBackendCalculator\_getMultilignCT(long, RNAstructureBackendCalculator, int) - Static method in class RNAstructure\_java\_interface.source.startup.RNAstructureBackendCalculatorProxyJNI


RNAstructureBackendCalculator\_getMultilignMaxPairs(long, RNAstructureBackendCalculator) - Static method in class RNAstructure\_java\_interface.source.startup.RNAstructureBackendCalculatorProxyJNI


RNAstructureBackendCalculator\_getMultilignSequenceSetData(long, RNAstructureBackendCalculator) - Static method in class RNAstructure\_java\_interface.source.startup.RNAstructureBackendCalculatorProxyJNI


RNAstructureBackendCalculator\_getNumMultilignSequences(long, RNAstructureBackendCalculator) - Static method in class RNAstructure\_java\_interface.source.startup.RNAstructureBackendCalculatorProxyJNI


RNAstructureBackendCalculator\_getNumTurboFoldSequences(long, RNAstructureBackendCalculator) - Static method in class RNAstructure\_java\_interface.source.startup.RNAstructureBackendCalculatorProxyJNI


RNAstructureBackendCalculator\_getOligoLabelData(long, RNAstructureBackendCalculator, int) - Static method in class RNAstructure\_java\_interface.source.startup.RNAstructureBackendCalculatorProxyJNI


RNAstructureBackendCalculator\_getOligoTargetLength(long, RNAstructureBackendCalculator) - Static method in class RNAstructure\_java\_interface.source.startup.RNAstructureBackendCalculatorProxyJNI


RNAstructureBackendCalculator\_getOligoTargetSequence(long, RNAstructureBackendCalculator) - Static method in class RNAstructure\_java\_interface.source.startup.RNAstructureBackendCalculatorProxyJNI


RNAstructureBackendCalculator\_getProgressNumber(long, RNAstructureBackendCalculator) - Static method in class RNAstructure\_java\_interface.source.startup.RNAstructureBackendCalculatorProxyJNI


RNAstructureBackendCalculator\_getRefoldWindowSize(long, RNAstructureBackendCalculator) - Static method in class RNAstructure\_java\_interface.source.startup.RNAstructureBackendCalculatorProxyJNI


RNAstructureBackendCalculator\_getSequenceComment(long, RNAstructureBackendCalculator) - Static method in class RNAstructure\_java\_interface.source.startup.RNAstructureBackendCalculatorProxyJNI


RNAstructureBackendCalculator\_getSequenceData(long, RNAstructureBackendCalculator) - Static method in class RNAstructure\_java\_interface.source.startup.RNAstructureBackendCalculatorProxyJNI


RNAstructureBackendCalculator\_getSequenceTitle(long, RNAstructureBackendCalculator) - Static method in class RNAstructure\_java\_interface.source.startup.RNAstructureBackendCalculatorProxyJNI


RNAstructureBackendCalculator\_getStructureType(long, RNAstructureBackendCalculator) - Static method in class RNAstructure\_java\_interface.source.startup.RNAstructureBackendCalculatorProxyJNI


RNAstructureBackendCalculator\_getSuboptimalAbsoluteDiff(long, RNAstructureBackendCalculator) - Static method in class RNAstructure\_java\_interface.source.startup.RNAstructureBackendCalculatorProxyJNI


RNAstructureBackendCalculator\_getSuboptimalPercentDiff(long, RNAstructureBackendCalculator) - Static method in class RNAstructure\_java\_interface.source.startup.RNAstructureBackendCalculatorProxyJNI


RNAstructureBackendCalculator\_getTemperature(long, RNAstructureBackendCalculator) - Static method in class RNAstructure\_java\_interface.source.startup.RNAstructureBackendCalculatorProxyJNI


RNAstructureBackendCalculator\_getTurboFoldCT(long, RNAstructureBackendCalculator, int) - Static method in class RNAstructure\_java\_interface.source.startup.RNAstructureBackendCalculatorProxyJNI


RNAstructureBackendCalculator\_getTurboFoldSaveFile(long, RNAstructureBackendCalculator, int) - Static method in class RNAstructure\_java\_interface.source.startup.RNAstructureBackendCalculatorProxyJNI


RNAstructureBackendCalculator\_getTurboFoldSequenceSetData(long, RNAstructureBackendCalculator) - Static method in class RNAstructure\_java\_interface.source.startup.RNAstructureBackendCalculatorProxyJNI


RNAstructureBackendCalculator\_readDynalignAlignmentConstraintsFile(long, RNAstructureBackendCalculator, String) - Static method in class RNAstructure\_java\_interface.source.startup.RNAstructureBackendCalculatorProxyJNI


RNAstructureBackendCalculator\_readFoldingConstraintsFile(long, RNAstructureBackendCalculator, String, int) - Static method in class RNAstructure\_java\_interface.source.startup.RNAstructureBackendCalculatorProxyJNI


RNAstructureBackendCalculator\_readSequenceData(long, RNAstructureBackendCalculator, String) - Static method in class RNAstructure\_java\_interface.source.startup.RNAstructureBackendCalculatorProxyJNI


RNAstructureBackendCalculator\_runAllSub(long, RNAstructureBackendCalculator, String, float, double) - Static method in class RNAstructure\_java\_interface.source.startup.RNAstructureBackendCalculatorProxyJNI


RNAstructureBackendCalculator\_runBifold(long, RNAstructureBackendCalculator, String, float, int, int, boolean, boolean) - Static method in class RNAstructure\_java\_interface.source.startup.RNAstructureBackendCalculatorProxyJNI


RNAstructureBackendCalculator\_runBipartition(long, RNAstructureBackendCalculator, String) - Static method in class RNAstructure\_java\_interface.source.startup.RNAstructureBackendCalculatorProxyJNI


RNAstructureBackendCalculator\_runDynalign(long, RNAstructureBackendCalculator, String, String, String, String, double, int, int, int, float, boolean) - Static method in class RNAstructure\_java\_interface.source.startup.RNAstructureBackendCalculatorProxyJNI


RNAstructureBackendCalculator\_runDynalignRefold(long, RNAstructureBackendCalculator, String, String, String, int, int, int, int) - Static method in class RNAstructure\_java\_interface.source.startup.RNAstructureBackendCalculatorProxyJNI


RNAstructureBackendCalculator\_runEfn2(long, RNAstructureBackendCalculator, String, boolean) - Static method in class RNAstructure\_java\_interface.source.startup.RNAstructureBackendCalculatorProxyJNI


RNAstructureBackendCalculator\_runFold(long, RNAstructureBackendCalculator, String, int, int, int, boolean) - Static method in class RNAstructure\_java\_interface.source.startup.RNAstructureBackendCalculatorProxyJNI


RNAstructureBackendCalculator\_runMaxExpect(long, RNAstructureBackendCalculator, String, double, int, int, double) - Static method in class RNAstructure\_java\_interface.source.startup.RNAstructureBackendCalculatorProxyJNI


RNAstructureBackendCalculator\_runMultilign(long, RNAstructureBackendCalculator, int, int, int, int, double, boolean, double, int, int, String, boolean, boolean) - Static method in class RNAstructure\_java\_interface.source.startup.RNAstructureBackendCalculatorProxyJNI


RNAstructureBackendCalculator\_runOligoScreen(long, RNAstructureBackendCalculator, String, String, boolean) - Static method in class RNAstructure\_java\_interface.source.startup.RNAstructureBackendCalculatorProxyJNI


RNAstructureBackendCalculator\_runOligoWalk(long, RNAstructureBackendCalculator, String, int, String, boolean, int, int, String, int, int) - Static method in class RNAstructure\_java\_interface.source.startup.RNAstructureBackendCalculatorProxyJNI


RNAstructureBackendCalculator\_runPartition(long, RNAstructureBackendCalculator, String) - Static method in class RNAstructure\_java\_interface.source.startup.RNAstructureBackendCalculatorProxyJNI


RNAstructureBackendCalculator\_runPseudoknotPrediction(long, RNAstructureBackendCalculator, String, int, int) - Static method in class RNAstructure\_java\_interface.source.startup.RNAstructureBackendCalculatorProxyJNI


RNAstructureBackendCalculator\_runPseudoknotRemoval(long, RNAstructureBackendCalculator, String, boolean) - Static method in class RNAstructure\_java\_interface.source.startup.RNAstructureBackendCalculatorProxyJNI


RNAstructureBackendCalculator\_runRefold(long, RNAstructureBackendCalculator, String, int, int, int) - Static method in class RNAstructure\_java\_interface.source.startup.RNAstructureBackendCalculatorProxyJNI


RNAstructureBackendCalculator\_runStochastic(long, RNAstructureBackendCalculator, String, int, int) - Static method in class RNAstructure\_java\_interface.source.startup.RNAstructureBackendCalculatorProxyJNI


RNAstructureBackendCalculator\_runTurboFoldMaximumExpectedAccuracy(long, RNAstructureBackendCalculator, double, int, double, int, int, double) - Static method in class RNAstructure\_java\_interface.source.startup.RNAstructureBackendCalculatorProxyJNI


RNAstructureBackendCalculator\_runTurboFoldPseudoknot(long, RNAstructureBackendCalculator, double, int, int, int) - Static method in class RNAstructure\_java\_interface.source.startup.RNAstructureBackendCalculatorProxyJNI


RNAstructureBackendCalculator\_runTurboFoldThreshold(long, RNAstructureBackendCalculator, double, int, double) - Static method in class RNAstructure\_java\_interface.source.startup.RNAstructureBackendCalculatorProxyJNI


RNAstructureBackendCalculator\_setCleavedNucleotide(long, RNAstructureBackendCalculator, int, int) - Static method in class RNAstructure\_java\_interface.source.startup.RNAstructureBackendCalculatorProxyJNI


RNAstructureBackendCalculator\_setDoubleStrandedNucleotide(long, RNAstructureBackendCalculator, int, int) - Static method in class RNAstructure\_java\_interface.source.startup.RNAstructureBackendCalculatorProxyJNI


RNAstructureBackendCalculator\_setDynalignAlignmentConstraint(long, RNAstructureBackendCalculator, int, int) - Static method in class RNAstructure\_java\_interface.source.startup.RNAstructureBackendCalculatorProxyJNI


RNAstructureBackendCalculator\_setForcedHelix(long, RNAstructureBackendCalculator, int, int, int, int) - Static method in class RNAstructure\_java\_interface.source.startup.RNAstructureBackendCalculatorProxyJNI


RNAstructureBackendCalculator\_setMaxLoop(long, RNAstructureBackendCalculator, int) - Static method in class RNAstructure\_java\_interface.source.startup.RNAstructureBackendCalculatorProxyJNI


RNAstructureBackendCalculator\_setMaxPair(long, RNAstructureBackendCalculator, int) - Static method in class RNAstructure\_java\_interface.source.startup.RNAstructureBackendCalculatorProxyJNI


RNAstructureBackendCalculator\_setModifiedNucleotide(long, RNAstructureBackendCalculator, int, int) - Static method in class RNAstructure\_java\_interface.source.startup.RNAstructureBackendCalculatorProxyJNI


RNAstructureBackendCalculator\_setProhibitedHelix(long, RNAstructureBackendCalculator, int, int, int, int) - Static method in class RNAstructure\_java\_interface.source.startup.RNAstructureBackendCalculatorProxyJNI


RNAstructureBackendCalculator\_setSequenceComment(long, RNAstructureBackendCalculator, String) - Static method in class RNAstructure\_java\_interface.source.startup.RNAstructureBackendCalculatorProxyJNI


RNAstructureBackendCalculator\_setSequenceData(long, RNAstructureBackendCalculator, String) - Static method in class RNAstructure\_java\_interface.source.startup.RNAstructureBackendCalculatorProxyJNI


RNAstructureBackendCalculator\_setSequenceTitle(long, RNAstructureBackendCalculator, String) - Static method in class RNAstructure\_java\_interface.source.startup.RNAstructureBackendCalculatorProxyJNI


RNAstructureBackendCalculator\_setSHAPEFile(long, RNAstructureBackendCalculator, String) - Static method in class RNAstructure\_java\_interface.source.startup.RNAstructureBackendCalculatorProxyJNI


RNAstructureBackendCalculator\_setSHAPEParam1(long, RNAstructureBackendCalculator, double) - Static method in class RNAstructure\_java\_interface.source.startup.RNAstructureBackendCalculatorProxyJNI


RNAstructureBackendCalculator\_setSHAPEParam2(long, RNAstructureBackendCalculator, double) - Static method in class RNAstructure\_java\_interface.source.startup.RNAstructureBackendCalculatorProxyJNI


RNAstructureBackendCalculator\_setSHAPEType(long, RNAstructureBackendCalculator, boolean) - Static method in class RNAstructure\_java\_interface.source.startup.RNAstructureBackendCalculatorProxyJNI


RNAstructureBackendCalculator\_setSingleStrandedNucleotide(long, RNAstructureBackendCalculator, int, int) - Static method in class RNAstructure\_java\_interface.source.startup.RNAstructureBackendCalculatorProxyJNI


RNAstructureBackendCalculator\_setTemperature(long, RNAstructureBackendCalculator, double) - Static method in class RNAstructure\_java\_interface.source.startup.RNAstructureBackendCalculatorProxyJNI


RNAstructureBackendCalculator\_writeDynalignAlignmentConstraintsFile(long, RNAstructureBackendCalculator, String) - Static method in class RNAstructure\_java\_interface.source.startup.RNAstructureBackendCalculatorProxyJNI


RNAstructureBackendCalculator\_writeFastaFile(long, RNAstructureBackendCalculator, String) - Static method in class RNAstructure\_java\_interface.source.startup.RNAstructureBackendCalculatorProxyJNI


RNAstructureBackendCalculator\_writeFoldingConstraintsFile(long, RNAstructureBackendCalculator, String, int) - Static method in class RNAstructure\_java\_interface.source.startup.RNAstructureBackendCalculatorProxyJNI


RNAstructureBackendCalculator\_writeSequenceFile(long, RNAstructureBackendCalculator, String) - Static method in class RNAstructure\_java\_interface.source.startup.RNAstructureBackendCalculatorProxyJNI


RNAstructureBackendCalculatorProxyJNI - Class in RNAstructure\_java\_interface.source.startup


RNAstructureBackendCalculatorProxyJNI() - Constructor for class RNAstructure\_java\_interface.source.startup.RNAstructureBackendCalculatorProxyJNI


RNAstructureMenu - Class in RNAstructure\_java\_utilities
:   A class that creates a menu.

RNAstructureMenu(String) - Constructor for class RNAstructure\_java\_utilities.RNAstructureMenu
:   Constructor.

rollover - Variable in class RNAstructure\_java\_interface.source.menus.RolloverMenu.RolloverListener
:   The rollover text for this listener.

RolloverMenu - Class in RNAstructure\_java\_interface.source.menus
:   A class that creates a menu for the RNAstructure GUI.

RolloverMenu(RNAstructureMenu) - Constructor for class RNAstructure\_java\_interface.source.menus.RolloverMenu
:   RNAstructureMenu Conversion Constructor.

RolloverMenu(String) - Constructor for class RNAstructure\_java\_interface.source.menus.RolloverMenu
:   Title Constructor.

RolloverMenu.RolloverListener - Class in RNAstructure\_java\_interface.source.menus
:   An inner class that creates a rollover listener for menus in the
    RNAstructure GUI.

RolloverMenu.RolloverListener(String) - Constructor for class RNAstructure\_java\_interface.source.menus.RolloverMenu.RolloverListener
:   Constructor.

runAllSub(String, float, double) - Method in class RNAstructure\_java\_interface.source.startup.RNAstructureBackendCalculator


runBifold(String, float, int, int, boolean, boolean) - Method in class RNAstructure\_java\_interface.source.startup.RNAstructureBackendCalculator


runBipartition(String) - Method in class RNAstructure\_java\_interface.source.startup.RNAstructureBackendCalculator


runDynalign(String, String, String, String, double, int, int, int, float, boolean) - Method in class RNAstructure\_java\_interface.source.startup.RNAstructureBackendCalculator


runDynalignRefold(String, String, String, int, int, int, int) - Method in class RNAstructure\_java\_interface.source.startup.RNAstructureBackendCalculator


runEfn2(String, boolean) - Method in class RNAstructure\_java\_interface.source.startup.RNAstructureBackendCalculator


runFold(String, int, int, int, boolean) - Method in class RNAstructure\_java\_interface.source.startup.RNAstructureBackendCalculator


runMainCalculation() - Method in class RNAstructure\_java\_interface.source.windows.DynalignFoldWindow


runMainCalculation() - Method in class RNAstructure\_java\_interface.source.windows.DynalignRefoldWindow


runMainCalculation() - Method in class RNAstructure\_java\_interface.source.windows.Efn2Window


runMainCalculation() - Method in class RNAstructure\_java\_interface.source.windows.FoldDoubleWindow


runMainCalculation() - Method in class RNAstructure\_java\_interface.source.windows.FoldSingleWindow


runMainCalculation() - Method in class RNAstructure\_java\_interface.source.windows.FoldSuboptimalWindow


runMainCalculation() - Method in class RNAstructure\_java\_interface.source.windows.MaxExpectWindow


runMainCalculation() - Method in class RNAstructure\_java\_interface.source.windows.ModuleWindow
:   Run the module calculation in the back end.

runMainCalculation() - Method in class RNAstructure\_java\_interface.source.windows.MultiWindow


runMainCalculation() - Method in class RNAstructure\_java\_interface.source.windows.OligoScreenWindow


runMainCalculation() - Method in class RNAstructure\_java\_interface.source.windows.OligoWalkWindow


runMainCalculation() - Method in class RNAstructure\_java\_interface.source.windows.PartitionDoubleWindow


runMainCalculation() - Method in class RNAstructure\_java\_interface.source.windows.PartitionSingleWindow


runMainCalculation() - Method in class RNAstructure\_java\_interface.source.windows.ProbKnotWindow


runMainCalculation() - Method in class RNAstructure\_java\_interface.source.windows.PseudoknotWindow


runMainCalculation() - Method in class RNAstructure\_java\_interface.source.windows.RefoldWindow


runMainCalculation() - Method in class RNAstructure\_java\_interface.source.windows.StochasticWindow


runMainCalculationMultipleSequences() - Method in class RNAstructure\_java\_interface.source.windows.MultilignWindow


runMainCalculationMultipleSequences() - Method in class RNAstructure\_java\_interface.source.windows.MultiWindow
:   Run the multiple sequence dependent module calculation in the back end.

runMainCalculationMultipleSequences() - Method in class RNAstructure\_java\_interface.source.windows.TurboFoldWindow


runMaxExpect(String, double, int, int, double) - Method in class RNAstructure\_java\_interface.source.startup.RNAstructureBackendCalculator


runMultilign(int, int, int, int, double, boolean, double, int, int, String, boolean, boolean) - Method in class RNAstructure\_java\_interface.source.startup.RNAstructureBackendCalculator


RunnerTemplate - Class in RNAstructure\_java\_drawing.source.runners
:   A class that templates the way in which the RNAstructure Java drawing
    framework is run independently.

RunnerTemplate(String) - Constructor for class RNAstructure\_java\_drawing.source.runners.RunnerTemplate
:   Constructor.

runOligoScreen(String, String, boolean) - Method in class RNAstructure\_java\_interface.source.startup.RNAstructureBackendCalculator


runOligoWalk(String, int, String, boolean, int, int, String, int, int) - Method in class RNAstructure\_java\_interface.source.startup.RNAstructureBackendCalculator


runPartition(String) - Method in class RNAstructure\_java\_interface.source.startup.RNAstructureBackendCalculator


runPseudoknotPrediction(String, int, int) - Method in class RNAstructure\_java\_interface.source.startup.RNAstructureBackendCalculator


runPseudoknotRemoval(String, boolean) - Method in class RNAstructure\_java\_interface.source.startup.RNAstructureBackendCalculator


runRefold(String, int, int, int) - Method in class RNAstructure\_java\_interface.source.startup.RNAstructureBackendCalculator


runStochastic(String, int, int) - Method in class RNAstructure\_java\_interface.source.startup.RNAstructureBackendCalculator


runTurboFoldMaximumExpectedAccuracy(double, int, double, int, int, double) - Method in class RNAstructure\_java\_interface.source.startup.RNAstructureBackendCalculator


runTurboFoldPseudoknot(double, int, int, int) - Method in class RNAstructure\_java\_interface.source.startup.RNAstructureBackendCalculator


runTurboFoldThreshold(double, int, double) - Method in class RNAstructure\_java\_interface.source.startup.RNAstructureBackendCalculator


## S

saveSequence() - Method in class RNAstructure\_java\_interface.source.windows.SequenceDisplayWindow
:   Save a sequence.

saveSequence(String) - Method in class RNAstructure\_java\_interface.source.windows.SequenceDisplayWindow
:   Save a sequence.

scale - Variable in class RNAstructure\_java\_drawing.source.dialogs.ImageDialog
:   The scale at which the image is seen.

scroll - Variable in class RNAstructure\_java\_interface.source.windows.MultiWindow
:   The scroll pane holding the sequence set.

ScrollerPane - Class in RNAstructure\_java\_utilities
:   A class that creates a scroll pane that can be controlled via the keyboard.

ScrollerPane(Component, int, int) - Constructor for class RNAstructure\_java\_utilities.ScrollerPane
:   Constructor.

select() - Method in class RNAstructure\_java\_interface.source.windows.OligoWalkWindow.BarButton
:   Select this button.

select(String, String) - Method in class RNAstructure\_java\_utilities.FileChooser
:   Select a file that has one possible input or output type.

select(String[], String[]) - Method in class RNAstructure\_java\_utilities.FileChooser
:   Select a file that has multiple possible input or output types.

selectFile() - Method in class RNAstructure\_java\_drawing.source.runners.PlotRunner


selectFile() - Method in class RNAstructure\_java\_drawing.source.runners.RunnerTemplate
:   Select an image file.

selectFile() - Method in class RNAstructure\_java\_drawing.source.runners.StructureRunner


selectionColor - Variable in class RNAstructure\_java\_interface.source.windows.OligoWalkWindow.BarButton
:   The selection color for this button.

selectorType - Variable in class RNAstructure\_java\_utilities.FileChooser
:   The type of file type selector.

SequenceDisplayWindow - Class in RNAstructure\_java\_interface.source.windows
:   A class responsible for displaying a window that allows a user to examine
    raw sequence data.

SequenceDisplayWindow() - Constructor for class RNAstructure\_java\_interface.source.windows.SequenceDisplayWindow
:   Default Constructor.

SequenceDisplayWindow(String) - Constructor for class RNAstructure\_java\_interface.source.windows.SequenceDisplayWindow
:   File Constructor.

SequenceDisplayWindow.EditMenu - Class in RNAstructure\_java\_interface.source.windows
:   An inner class that creates a sequence editing menu.

SequenceDisplayWindow.EditMenu() - Constructor for class RNAstructure\_java\_interface.source.windows.SequenceDisplayWindow.EditMenu
:   Constructor.

SequenceDisplayWindow.ReadMenu - Class in RNAstructure\_java\_interface.source.windows
:   An inner class that creates a sequence reading menu.

SequenceDisplayWindow.ReadMenu() - Constructor for class RNAstructure\_java\_interface.source.windows.SequenceDisplayWindow.ReadMenu
:   Constructor.

serialVersionUID - Static variable in class RNAstructure\_java\_drawing.source.dialogs.ImageDialog.PrintMenu


serialVersionUID - Static variable in class RNAstructure\_java\_drawing.source.dialogs.ImageDialog


serialVersionUID - Static variable in class RNAstructure\_java\_drawing.source.dialogs.ImageDialog.ZoomDialog


serialVersionUID - Static variable in class RNAstructure\_java\_drawing.source.dialogs.PlotDialog


serialVersionUID - Static variable in class RNAstructure\_java\_drawing.source.dialogs.StructureDialog


serialVersionUID - Static variable in class RNAstructure\_java\_drawing.source.menus.AnnotationMenu


serialVersionUID - Static variable in class RNAstructure\_java\_drawing.source.menus.DotsMenu.ColorDialog


serialVersionUID - Static variable in class RNAstructure\_java\_drawing.source.menus.DotsMenu.RangeDialog


serialVersionUID - Static variable in class RNAstructure\_java\_drawing.source.menus.DotsMenu


serialVersionUID - Static variable in class RNAstructure\_java\_drawing.source.menus.PlotMenu


serialVersionUID - Static variable in class RNAstructure\_java\_drawing.source.menus.StructureMenu.MoveDialog


serialVersionUID - Static variable in class RNAstructure\_java\_drawing.source.menus.StructureMenu


serialVersionUID - Static variable in class RNAstructure\_java\_drawing.source.runners.PlotRunner


serialVersionUID - Static variable in class RNAstructure\_java\_drawing.source.runners.StructureRunner


serialVersionUID - Static variable in class RNAstructure\_java\_interface.source.menus.ConstraintsMenu.NucOrHelixDialog


serialVersionUID - Static variable in class RNAstructure\_java\_interface.source.menus.ConstraintsMenu


serialVersionUID - Static variable in class RNAstructure\_java\_interface.source.menus.DynamicMenuBar


serialVersionUID - Static variable in class RNAstructure\_java\_interface.source.menus.FileMenu


serialVersionUID - Static variable in class RNAstructure\_java\_interface.source.menus.HelpMenu


serialVersionUID - Static variable in class RNAstructure\_java\_interface.source.menus.NucleicAcidMenu


serialVersionUID - Static variable in class RNAstructure\_java\_interface.source.menus.RolloverMenu.RolloverListener


serialVersionUID - Static variable in class RNAstructure\_java\_interface.source.menus.RolloverMenu


serialVersionUID - Static variable in class RNAstructure\_java\_interface.source.startup.ApplicationRootFrame


serialVersionUID - Static variable in class RNAstructure\_java\_interface.source.startup.RNAstructure


serialVersionUID - Static variable in class RNAstructure\_java\_interface.source.windows.DrawingWindow


serialVersionUID - Static variable in class RNAstructure\_java\_interface.source.windows.DynalignFoldWindow


serialVersionUID - Static variable in class RNAstructure\_java\_interface.source.windows.DynalignRefoldWindow


serialVersionUID - Static variable in class RNAstructure\_java\_interface.source.windows.Efn2Window


serialVersionUID - Static variable in class RNAstructure\_java\_interface.source.windows.FoldDoubleWindow


serialVersionUID - Static variable in class RNAstructure\_java\_interface.source.windows.FoldSingleWindow


serialVersionUID - Static variable in class RNAstructure\_java\_interface.source.windows.FoldSuboptimalWindow


serialVersionUID - Static variable in class RNAstructure\_java\_interface.source.windows.InternalWindow


serialVersionUID - Static variable in class RNAstructure\_java\_interface.source.windows.MaxExpectWindow


serialVersionUID - Static variable in class RNAstructure\_java\_interface.source.windows.ModuleWindow


serialVersionUID - Static variable in class RNAstructure\_java\_interface.source.windows.MultilignWindow


serialVersionUID - Static variable in class RNAstructure\_java\_interface.source.windows.MultiWindow


serialVersionUID - Static variable in class RNAstructure\_java\_interface.source.windows.OligoScreenWindow


serialVersionUID - Static variable in class RNAstructure\_java\_interface.source.windows.OligoWalkWindow.BarButton


serialVersionUID - Static variable in class RNAstructure\_java\_interface.source.windows.OligoWalkWindow.OligoSelectionDialog


serialVersionUID - Static variable in class RNAstructure\_java\_interface.source.windows.OligoWalkWindow.OligoWalkResultsMenu


serialVersionUID - Static variable in class RNAstructure\_java\_interface.source.windows.OligoWalkWindow


serialVersionUID - Static variable in class RNAstructure\_java\_interface.source.windows.OligoWalkWindow.SpinnerPanel


serialVersionUID - Static variable in class RNAstructure\_java\_interface.source.windows.OligoWalkWindow.StringComboBox


serialVersionUID - Static variable in class RNAstructure\_java\_interface.source.windows.PartitionDoubleWindow


serialVersionUID - Static variable in class RNAstructure\_java\_interface.source.windows.PartitionSingleWindow


serialVersionUID - Static variable in class RNAstructure\_java\_interface.source.windows.ProbKnotWindow


serialVersionUID - Static variable in class RNAstructure\_java\_interface.source.windows.PseudoknotWindow


serialVersionUID - Static variable in class RNAstructure\_java\_interface.source.windows.RefoldWindow


serialVersionUID - Static variable in class RNAstructure\_java\_interface.source.windows.SequenceDisplayWindow.EditMenu


serialVersionUID - Static variable in class RNAstructure\_java\_interface.source.windows.SequenceDisplayWindow.ReadMenu


serialVersionUID - Static variable in class RNAstructure\_java\_interface.source.windows.SequenceDisplayWindow


serialVersionUID - Static variable in class RNAstructure\_java\_interface.source.windows.StochasticWindow


serialVersionUID - Static variable in class RNAstructure\_java\_interface.source.windows.TurboFoldWindow


serialVersionUID - Static variable in class RNAstructure\_java\_utilities.BorderBuilder


serialVersionUID - Static variable in class RNAstructure\_java\_utilities.BorderedButtonPanel


serialVersionUID - Static variable in class RNAstructure\_java\_utilities.FieldPanel.FilePanel


serialVersionUID - Static variable in class RNAstructure\_java\_utilities.FieldPanel.NumberPanel


serialVersionUID - Static variable in class RNAstructure\_java\_utilities.FieldPanel


serialVersionUID - Static variable in class RNAstructure\_java\_utilities.FileChooser


serialVersionUID - Static variable in class RNAstructure\_java\_utilities.FileField


serialVersionUID - Static variable in class RNAstructure\_java\_utilities.HTMLCheckBox


serialVersionUID - Static variable in class RNAstructure\_java\_utilities.ImageGrabber


serialVersionUID - Static variable in class RNAstructure\_java\_utilities.MacChecker


serialVersionUID - Static variable in class RNAstructure\_java\_utilities.NumberField.DoubleField


serialVersionUID - Static variable in class RNAstructure\_java\_utilities.NumberField.FloatField


serialVersionUID - Static variable in class RNAstructure\_java\_utilities.NumberField.IntegerField


serialVersionUID - Static variable in class RNAstructure\_java\_utilities.NumberField


serialVersionUID - Static variable in class RNAstructure\_java\_utilities.RadioButtonPanel


serialVersionUID - Static variable in class RNAstructure\_java\_utilities.RNAstructureMenu


serialVersionUID - Static variable in class RNAstructure\_java\_utilities.ScrollerPane


serialVersionUID - Static variable in class RNAstructure\_java\_utilities.SimpleDialogHandler


serialVersionUID - Static variable in class RNAstructure\_java\_utilities.ValueSelectionDialog


set() - Method in class RNAstructure\_java\_interface.source.menus.RolloverMenu.RolloverListener
:   Set the main frame message bar text to this listener's specific
    rollover text.

setAnchorCenter() - Method in class RNAstructure\_java\_interface.source.windows.ModuleWindow
:   Set the anchoring area for the GridBagConstraints to CENTER.

setAnchorNorth() - Method in class RNAstructure\_java\_interface.source.windows.ModuleWindow
:   Set the anchoring area for the GridBagConstraints to NORTH.

setAnnotation(String) - Method in class RNAstructure\_java\_drawing.source.dialogs.StructureDialog
:   Set the annotation for this structure drawing.

setBarColor(Color) - Method in class RNAstructure\_java\_interface.source.windows.OligoWalkWindow.BarButton
:   Set the default color that will be painted on this button.

setBarPolicies(int, int) - Method in class RNAstructure\_java\_utilities.ScrollerPane
:   Set the scroll bar policies for this scroll pane.

setCleavedNucleotide(int, int) - Method in class RNAstructure\_java\_interface.source.startup.RNAstructureBackendCalculator


setColors(int) - Method in class RNAstructure\_java\_drawing.source.dialogs.PlotDialog
:   Set the number of colors in the plot on this dialog.

setColors(int) - Method in class RNAstructure\_java\_drawing.source.proxy.DotPlotBackend


setConstraint(String) - Method in class RNAstructure\_java\_interface.source.menus.ConstraintsMenu.NucOrHelixDialog
:   Set a constraint using this dialog.

setDataAutomatically(String) - Method in class RNAstructure\_java\_interface.source.windows.FoldSingleWindow
:   Set the window's data automatically.

setDoubleStrandedNucleotide(int, int) - Method in class RNAstructure\_java\_interface.source.startup.RNAstructureBackendCalculator


setDynalignAlignmentConstraint(int, int) - Method in class RNAstructure\_java\_interface.source.startup.RNAstructureBackendCalculator


setFile(int, String) - Method in class RNAstructure\_java\_utilities.FieldPanel.FilePanel
:   Set a file name in a particular input field.

setFillCenter() - Method in class RNAstructure\_java\_interface.source.windows.ModuleWindow
:   Set the fill for the GridBagConstraints as CENTER.

setFillHorizontal() - Method in class RNAstructure\_java\_interface.source.windows.ModuleWindow
:   Set the fill for the GridBagConstraints as HORIZONTAL.

setForcedHelix(int, int, int, int) - Method in class RNAstructure\_java\_interface.source.startup.RNAstructureBackendCalculator


setGrid(int, int) - Method in class RNAstructure\_java\_interface.source.windows.ModuleWindow
:   Set the amount of space a component takes up in the grid.

setInfoLabel(String) - Method in class RNAstructure\_java\_interface.source.startup.ApplicationRootFrame
:   Set the info bar at the bottom of the main window to contain the
    specified text.

setInsets(int, int, int, int) - Method in class RNAstructure\_java\_interface.source.windows.ModuleWindow
:   Set the external padding of the constraints.

setMaxLoop(int) - Method in class RNAstructure\_java\_interface.source.startup.RNAstructureBackendCalculator


setMaxPair(int) - Method in class RNAstructure\_java\_interface.source.startup.RNAstructureBackendCalculator


setMenus() - Method in class RNAstructure\_java\_interface.source.windows.DrawingWindow
:   Create the array of variable menus used by this window.

setMenus() - Method in class RNAstructure\_java\_interface.source.windows.DynalignFoldWindow


setMenus() - Method in class RNAstructure\_java\_interface.source.windows.DynalignRefoldWindow


setMenus() - Method in class RNAstructure\_java\_interface.source.windows.Efn2Window


setMenus() - Method in class RNAstructure\_java\_interface.source.windows.FoldDoubleWindow


setMenus() - Method in class RNAstructure\_java\_interface.source.windows.FoldSingleWindow


setMenus() - Method in class RNAstructure\_java\_interface.source.windows.FoldSuboptimalWindow


setMenus() - Method in class RNAstructure\_java\_interface.source.windows.InternalWindow
:   Create the array of variable menus used by this window.

setMenus() - Method in class RNAstructure\_java\_interface.source.windows.MaxExpectWindow


setMenus() - Method in class RNAstructure\_java\_interface.source.windows.MultiWindow


setMenus() - Method in class RNAstructure\_java\_interface.source.windows.OligoScreenWindow


setMenus() - Method in class RNAstructure\_java\_interface.source.windows.OligoWalkWindow


setMenus() - Method in class RNAstructure\_java\_interface.source.windows.PartitionDoubleWindow


setMenus() - Method in class RNAstructure\_java\_interface.source.windows.PartitionSingleWindow


setMenus() - Method in class RNAstructure\_java\_interface.source.windows.ProbKnotWindow


setMenus() - Method in class RNAstructure\_java\_interface.source.windows.PseudoknotWindow


setMenus() - Method in class RNAstructure\_java\_interface.source.windows.RefoldWindow


setMenus() - Method in class RNAstructure\_java\_interface.source.windows.SequenceDisplayWindow


setMenus() - Method in class RNAstructure\_java\_interface.source.windows.StochasticWindow


setModifiedNucleotide(int, int) - Method in class RNAstructure\_java\_interface.source.startup.RNAstructureBackendCalculator


setNucleotidesCircled(boolean) - Method in class RNAstructure\_java\_drawing.source.proxy.StructureBackend


setOligoData(int) - Method in class RNAstructure\_java\_interface.source.windows.OligoWalkWindow
:   Set the data for a particular oligo.

setPad(int, int) - Method in class RNAstructure\_java\_interface.source.windows.ModuleWindow
:   Set the internal padding of the constraints.

setPanelWidth(int) - Method in class RNAstructure\_java\_utilities.FieldPanel
:   Set the width of the panel.

setProhibitedHelix(int, int, int, int) - Method in class RNAstructure\_java\_interface.source.startup.RNAstructureBackendCalculator


setRange(double, double) - Method in class RNAstructure\_java\_drawing.source.dialogs.PlotDialog
:   Set the range of the plot on this dialog.

setRange(double, double) - Method in class RNAstructure\_java\_drawing.source.proxy.DotPlotBackend


setReading(boolean) - Method in class RNAstructure\_java\_interface.source.windows.SequenceDisplayWindow
:   Set whether or not the window should be reading while typing.

setScale(int) - Method in class RNAstructure\_java\_drawing.source.dialogs.ImageDialog
:   Set the image scale.

setSelectedButton(String) - Method in class RNAstructure\_java\_utilities.RadioButtonPanel
:   Set a particular button in this group to be selected.

setSequenceComment(String) - Method in class RNAstructure\_java\_interface.source.startup.RNAstructureBackendCalculator


setSequenceData(String) - Method in class RNAstructure\_java\_interface.source.startup.RNAstructureBackendCalculator


setSequenceTitle(String) - Method in class RNAstructure\_java\_interface.source.startup.RNAstructureBackendCalculator


setSHAPEFile(String) - Method in class RNAstructure\_java\_interface.source.startup.RNAstructureBackendCalculator


setSHAPEParam1(double) - Method in class RNAstructure\_java\_interface.source.startup.RNAstructureBackendCalculator


setSHAPEParam2(double) - Method in class RNAstructure\_java\_interface.source.startup.RNAstructureBackendCalculator


setSHAPEType(boolean) - Method in class RNAstructure\_java\_interface.source.startup.RNAstructureBackendCalculator


setSingleStrandedNucleotide(int, int) - Method in class RNAstructure\_java\_interface.source.startup.RNAstructureBackendCalculator


setSizes(int, int) - Method in class RNAstructure\_java\_utilities.ScrollerPane
:   Set the size of the scroller pane by setting preferred, minimum, and
    maximum sizes all at once.

setStructureNumber(int) - Method in class RNAstructure\_java\_drawing.source.dialogs.StructureDialog
:   Set the structure number.

setTemperature(double) - Method in class RNAstructure\_java\_interface.source.startup.RNAstructureBackendCalculator


setTitles(String) - Method in class RNAstructure\_java\_interface.source.windows.InternalWindow
:   Set the title of both this window and the main window.

setTopCaption(String) - Method in class RNAstructure\_java\_drawing.source.dialogs.ImageDialog
:   Set the text on the info label.

showDeletionConfirmation() - Method in class RNAstructure\_java\_interface.source.menus.ConstraintsMenu
:   Show a dialog that asks whether constraints should be deleted.

showImageDialogs() - Method in class RNAstructure\_java\_interface.source.windows.MultiWindow
:   Show the image dialogs, if possible.

showProgressBar(boolean) - Method in class RNAstructure\_java\_interface.source.windows.ModuleWindow
:   Create a progress bar.

showProgressBarDeterminate() - Method in class RNAstructure\_java\_interface.source.windows.ModuleWindow
:   Create a determinate progress bar.

showProgressBarIndeterminate() - Method in class RNAstructure\_java\_interface.source.windows.ModuleWindow
:   Create an indeterminate progress bar.

SimpleDialogHandler - Class in RNAstructure\_java\_utilities
:   A class that handles simple dialogs used in the RNAstructure GUI.

SimpleDialogHandler() - Constructor for class RNAstructure\_java\_utilities.SimpleDialogHandler


specificPanel - Variable in class RNAstructure\_java\_interface.source.windows.TurboFoldWindow
:   The panel that holds the dynamic options applicable to a mode.

specifiedPanelWidth - Variable in class RNAstructure\_java\_utilities.FieldPanel
:   The specified width of the panel.

spinner - Variable in class RNAstructure\_java\_interface.source.windows.OligoWalkWindow.SpinnerPanel
:   The spinner in this panel.

StochasticWindow - Class in RNAstructure\_java\_interface.source.windows
:   A class responsible for initializing and running the stochastic module,
    which generates a stochastic sample of structures.

StochasticWindow(String) - Constructor for class RNAstructure\_java\_interface.source.windows.StochasticWindow
:   Constructor.

str - Variable in class RNAstructure\_java\_interface.source.menus.ConstraintsMenu
:   The strand this constraints menu deals with.

strand - Variable in class RNAstructure\_java\_drawing.source.dialogs.PlotDialog
:   The strand being drawn.

strand - Variable in class RNAstructure\_java\_interface.source.windows.DrawingWindow
:   The strand being drawn.

StructureBackend - Class in RNAstructure\_java\_drawing.source.proxy


StructureBackend(long, boolean) - Constructor for class RNAstructure\_java\_drawing.source.proxy.StructureBackend


StructureBackend() - Constructor for class RNAstructure\_java\_drawing.source.proxy.StructureBackend


StructureBackend\_addAnnotationProbability(long, StructureBackend, String) - Static method in class RNAstructure\_java\_drawing.source.proxy.StructureBackendProxyJNI


StructureBackend\_addAnnotationSHAPE(long, StructureBackend, String) - Static method in class RNAstructure\_java\_drawing.source.proxy.StructureBackendProxyJNI


StructureBackend\_flip(long, StructureBackend) - Static method in class RNAstructure\_java\_drawing.source.proxy.StructureBackendProxyJNI


StructureBackend\_getStructureData(long, StructureBackend, int) - Static method in class RNAstructure\_java\_drawing.source.proxy.StructureBackendProxyJNI


StructureBackend\_readStructureData(long, StructureBackend, String) - Static method in class RNAstructure\_java\_drawing.source.proxy.StructureBackendProxyJNI


StructureBackend\_removeAnnotation(long, StructureBackend) - Static method in class RNAstructure\_java\_drawing.source.proxy.StructureBackendProxyJNI


StructureBackend\_setNucleotidesCircled(long, StructureBackend, boolean) - Static method in class RNAstructure\_java\_drawing.source.proxy.StructureBackendProxyJNI


StructureBackend\_writeDotBracketFile(long, StructureBackend, String, String) - Static method in class RNAstructure\_java\_drawing.source.proxy.StructureBackendProxyJNI


StructureBackend\_writeHelixFile(long, StructureBackend, String, String, int) - Static method in class RNAstructure\_java\_drawing.source.proxy.StructureBackendProxyJNI


StructureBackend\_writePostscriptFile(long, StructureBackend, String, int) - Static method in class RNAstructure\_java\_drawing.source.proxy.StructureBackendProxyJNI


StructureBackend\_writeSVGFile(long, StructureBackend, String, int) - Static method in class RNAstructure\_java\_drawing.source.proxy.StructureBackendProxyJNI


StructureBackendProxyJNI - Class in RNAstructure\_java\_drawing.source.proxy


StructureBackendProxyJNI() - Constructor for class RNAstructure\_java\_drawing.source.proxy.StructureBackendProxyJNI


StructureDialog - Class in RNAstructure\_java\_drawing.source.dialogs
:   A dialog that holds structure images.

StructureDialog(String) - Constructor for class RNAstructure\_java\_drawing.source.dialogs.StructureDialog
:   Constructor.

StructureMenu - Class in RNAstructure\_java\_drawing.source.menus
:   A class that handles a menu which can manipulate a drawn structure.

StructureMenu(StructureDialog) - Constructor for class RNAstructure\_java\_drawing.source.menus.StructureMenu
:   Constructor.

StructureMenu.MoveDialog - Class in RNAstructure\_java\_drawing.source.menus
:   An inner class which creates a dialog that moves to a structure.

StructureMenu.MoveDialog() - Constructor for class RNAstructure\_java\_drawing.source.menus.StructureMenu.MoveDialog
:   Constructor.

StructureRunner - Class in RNAstructure\_java\_drawing.source.runners
:   A class that runs RNAstructure Java structure drawing independently of the
    main RNAstructure application.

StructureRunner() - Constructor for class RNAstructure\_java\_drawing.source.runners.StructureRunner
:   Constructor.

swigCMemOwn - Variable in class RNAstructure\_java\_drawing.source.proxy.DotPlotBackend


swigCMemOwn - Variable in class RNAstructure\_java\_drawing.source.proxy.StructureBackend


swigCMemOwn - Variable in class RNAstructure\_java\_interface.source.startup.RNAstructureBackendCalculator


swigCPtr - Variable in class RNAstructure\_java\_drawing.source.proxy.DotPlotBackend


swigCPtr - Variable in class RNAstructure\_java\_drawing.source.proxy.StructureBackend


swigCPtr - Variable in class RNAstructure\_java\_interface.source.startup.RNAstructureBackendCalculator


switchCircled() - Method in class RNAstructure\_java\_drawing.source.dialogs.StructureDialog
:   Switch circled nucleotides on or off.


## T

textFields - Variable in class RNAstructure\_java\_utilities.FieldPanel
:   The array of text fields.

timer - Variable in class RNAstructure\_java\_interface.source.windows.SequenceDisplayWindow
:   The timer that aids in reading nucleotides in rhythm.

TurboFoldWindow - Class in RNAstructure\_java\_interface.source.windows
:   A class responsible for initializing and running the TurboFold module,
    which calculates common secondary structures of multiple sequences using
    the partition function and probabalistic alignments.

TurboFoldWindow() - Constructor for class RNAstructure\_java\_interface.source.windows.TurboFoldWindow
:   Constructor.


## V

valueOf(String) - Static method in enum RNAstructure\_java\_utilities.FileChooser.Types
:   Returns the enum constant of this type with the specified name.

values() - Static method in enum RNAstructure\_java\_utilities.FileChooser.Types
:   Returns an array containing the constants of this enum type, in
    the order they are declared.

ValueSelectionDialog - Class in RNAstructure\_java\_utilities
:   A class which creates a dialog that selects values from labeled text fields.

ValueSelectionDialog(String) - Constructor for class RNAstructure\_java\_utilities.ValueSelectionDialog
:   Constructor.

viewDialog() - Method in class RNAstructure\_java\_drawing.source.dialogs.ImageDialog
:   View the dialog.

viewWindow() - Method in class RNAstructure\_java\_interface.source.windows.InternalWindow
:   View the window.

viewZoomDialog() - Method in class RNAstructure\_java\_drawing.source.dialogs.ImageDialog
:   View a zooming dialog.


## W

writeDotBracketFile(String) - Method in class RNAstructure\_java\_drawing.source.dialogs.StructureDialog
:   Write a dot bracket file of all structures on this dialog.

writeDotBracketFile(String, String) - Method in class RNAstructure\_java\_drawing.source.proxy.StructureBackend


writeDynalignAlignmentConstraintsFile(String) - Method in class RNAstructure\_java\_interface.source.startup.RNAstructureBackendCalculator


writeFastaFile(String) - Method in class RNAstructure\_java\_interface.source.startup.RNAstructureBackendCalculator


writeFoldingConstraintsFile(String, int) - Method in class RNAstructure\_java\_interface.source.startup.RNAstructureBackendCalculator


writeHelixFile(String) - Method in class RNAstructure\_java\_drawing.source.dialogs.StructureDialog
:   Write a helix file from the current structure on this dialog.

writeHelixFile(String, String, int) - Method in class RNAstructure\_java\_drawing.source.proxy.StructureBackend


writePostscriptFile(String) - Method in class RNAstructure\_java\_drawing.source.dialogs.PlotDialog
:   Write a Postscript file from the plot on this dialog.

writePostscriptFile(String) - Method in class RNAstructure\_java\_drawing.source.dialogs.StructureDialog
:   Write a Postscript file from the current structure on this dialog.

writePostscriptFile(String) - Method in class RNAstructure\_java\_drawing.source.proxy.DotPlotBackend


writePostscriptFile(String, int) - Method in class RNAstructure\_java\_drawing.source.proxy.StructureBackend


writeSequenceFile(String) - Method in class RNAstructure\_java\_interface.source.startup.RNAstructureBackendCalculator


writeStructuresFile(String, boolean) - Method in class RNAstructure\_java\_drawing.source.dialogs.PlotDialog
:   Write a probable structures file from the plot on this dialog.

writeStructuresFile(String, String) - Method in class RNAstructure\_java\_drawing.source.proxy.DotPlotBackend


writeSVGFile(String) - Method in class RNAstructure\_java\_drawing.source.dialogs.PlotDialog
:   Write an SVG file from the plot on this dialog.

writeSVGFile(String) - Method in class RNAstructure\_java\_drawing.source.dialogs.StructureDialog
:   Write an SVG file from the current structure on this dialog.

writeSVGFile(String) - Method in class RNAstructure\_java\_drawing.source.proxy.DotPlotBackend


writeSVGFile(String, int) - Method in class RNAstructure\_java\_drawing.source.proxy.StructureBackend


writeTextFile(String) - Method in class RNAstructure\_java\_drawing.source.dialogs.PlotDialog
:   Write a text file from the plot on this dialog.

writeTextFile(String) - Method in class RNAstructure\_java\_drawing.source.proxy.DotPlotBackend


## Z

zoomImage() - Method in class RNAstructure\_java\_drawing.source.dialogs.ImageDialog
:   Zoom the image on the panel.

A B C D E F G H I J L M N O P R S T V W Z

- Overview
- Package
- Class
- Tree
- Index
- Help

- Prev
- Next

- Frames
- No Frames

- All Classes
